# Optimization of injection molding parameters for bending and tensile strength of ABS and ABS/carbon fiber composites using Taguchi–Grey correlation analysis

**DOI:** 10.1371/journal.pone.0352647

**Published:** 2026-07-15

**Authors:** Hieu Giang Le, Thanh Trung Do, Tran Minh The Uyen, Bui Chan Thanh, Pham Son Minh

**Affiliations:** Faculty of Mechanical Engineering, Ho Chi Minh City University of Technology and Engineering, Ho Chi Minh City, Vietnam; King Mongkut’s University of Technology North Bangkok, THAILAND

## Abstract

This study investigates the effects of injection molding parameters on the flexural and tensile strengths of neat acrylonitrile–butadiene–styrene (ABS) and woven carbon fiber fabric–reinforced ABS composites (ABS/CF) fabricated by a two-step insert injection molding process. A Taguchi L25 orthogonal experimental design was employed with five processing parameters: filling pressure (104–118 MPa), packing pressure (96–100 MPa), filling time (1–3 s), packing time (0.5–2.5 s), and melt temperature (228–232 °C). The results show that flexural strength increased from 5.86–6.49 MPa (ABS) to 5.76–6.93 MPa (ABS/CF), while tensile strength increased from 33.35–35.17 MPa to 38.20–41.27 MPa, corresponding to improvements of approximately 7% and 17–18%, respectively. ANOVA results indicate that filling pressure is the most significant factor, with p-values of 0.012 and 0.039 for flexural performance and a maximum contribution of 47.08% for tensile strength. The optimal parameter combination identified by Taguchi–Grey analysis yields Grey relational grades of 0.725 and 0.728 for flexural and tensile strength, respectively. Confirmation experiments show good agreement with predicted values, with deviations of 1.59% (flexural) and 2.98% (tensile). Overall, this study provides a comprehensive understanding of the process–structure–property relationships in injection-molded ABS/CF composites and demonstrates that the Taguchi–Grey approach is an effective method for simultaneous optimization of multiple mechanical properties. The findings offer practical guidelines for improving the mechanical performance of fiber-reinforced thermoplastic composites in thin-walled and structural applications.

## 1. Introduction

Carbon fiber–reinforced polymer composites have been widely adopted in engineering applications due to their high specific strength, stiffness, and design flexibility [1–2]. In recent years, thermoplastic-based composites have attracted increasing attention compared to conventional thermoset systems because of their shorter processing cycles, recyclability, and compatibility with high-throughput manufacturing technologies such as injection molding [3].

Among engineering thermoplastics, acrylonitrile butadiene styrene (ABS) is extensively used in industrial applications owing to its balanced mechanical properties, impact resistance, and processability [4]. However, the relatively low stiffness and load-bearing capacity of neat ABS limit its application in structural and thin-walled components subjected to significant bending and tensile loads. To address these limitations, numerous studies have investigated the reinforcement of ABS using carbon fibers, primarily in the form of short or chopped fibers [5,6]. These studies consistently report improvements in tensile strength and modulus; however, the reinforcement efficiency remains limited due to random fiber orientation, fiber breakage during processing, and insufficient stress transfer at the fiber–matrix interface [7].

To overcome these limitations, recent studies have increasingly focused on continuous and woven carbon fiber reinforcements, which provide more effective load transfer and improved structural integrity compared to short fiber systems [8]. In particular, woven carbon fabrics such as Plain Weave 3K offer bidirectional reinforcement, enabling more uniform stress distribution and enhanced mechanical performance. Nevertheless, most of these studies are restricted to thermoset-based composites fabricated via processes such as resin transfer molding (RTM), vacuum-assisted resin infusion (VARI), or compression molding [9], with limited attention to thermoplastic injection molding systems.

For thermoplastic-based composites, especially those processed by injection molding, recent findings indicate that processing parameters play a critical role in determining fiber impregnation quality, interfacial bonding, and final mechanical performance [10–11]. Improper parameter selection may lead to defects such as void formation, incomplete filling, weak interfacial bonding, and non-uniform fiber distribution, which significantly degrade the mechanical properties of the final products. These challenges are further exacerbated in thin-walled components due to rapid cooling and high flow resistance.

Processing parameters in injection molding, including filling pressure, packing pressure, filling time, packing time, and melt temperature, govern both the flow behavior of the molten polymer and the resulting microstructure of the composite. Recent studies have demonstrated that filling pressure strongly influences fiber wetting and mold filling behavior, while packing pressure contributes to material densification and void reduction [12–14]. In addition, melt temperature affects polymer viscosity and flowability, thereby influencing fiber impregnation and interfacial bonding [15,16]. Recent experimental studies have reported that increasing packing pressure can improve the tensile strength of thermoplastic composites by approximately 15–25%, primarily due to enhanced material densification and reduced void content. Similarly, higher melt temperatures have been shown to reduce polymer viscosity and improve fiber impregnation, leading to improvements in interfacial bonding and mechanical strength of up to 10–20% in fiber-reinforced systems. In addition, optimized filling pressure has been found to significantly enhance mold filling quality and fiber wetting, thereby reducing defects such as incomplete impregnation and weak interfacial regions, which are critical factors affecting the final mechanical performance. However, most existing studies focus on single-response optimization or short fiber–reinforced systems, and comprehensive investigations on woven fabric–reinforced thermoplastic composites remain scarce.

More importantly, the combined and interactive effects of multiple injection molding parameters on the mechanical performance of ABS composites reinforced with woven carbon fabrics—particularly under thin-walled conditions—are still not well understood. Furthermore, there is a lack of studies applying multi-objective optimization approaches to simultaneously optimize both tensile and flexural properties for this material system. This represents a critical research gap in the current literature.

To address this gap, advanced statistical approaches such as the Taguchi method combined with Grey Relational Analysis (GRA) and Analysis of Variance (ANOVA) have been widely applied for multi-response optimization in manufacturing processes [17–21]. Furthermore, optimization methods such as Taguchi, Response Surface Methodology (RSM), and Machine Learning (ML) have been successfully applied across diverse engineering fields. These methods have proven highly effective in modeling wear behavior, optimizing machining parameters, and predicting the mechanical properties of various hybrid composite materials [22–27]. However, the application of the TGC method to injection-molded ABS/woven carbon fabric composites remains limited.

**Fig 1:**
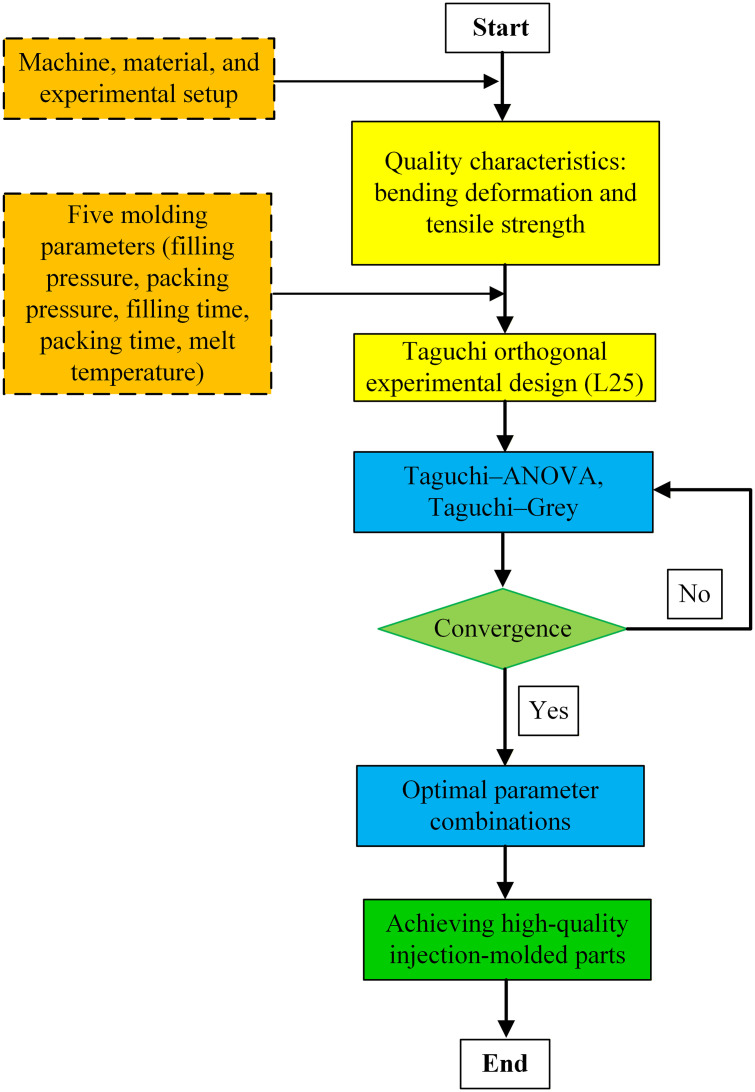
Flowchart of the optimization procedure for injection molding parameters using Taguchi orthogonal experimental design (L25) combined with Taguchi–ANOVA and Taguchi–Grey methods to minimize bending deformation and maximize tensile strength.

Therefore, the present study aims to systematically investigate the combined effects of key injection molding parameters—filling pressure, packing pressure, filling time, packing time, and melt temperature—on both flexural and tensile strength of neat ABS and ABS composites reinforced with Plain Weave 3K carbon fabric. A Taguchi L25 orthogonal array is employed to construct the experimental design, while Taguchi–Grey relational analysis is used for multi-objective optimization, and ANOVA is applied to evaluate the statistical significance and contribution of each parameter. The outcomes of this study provide both a clearer understanding of the process–structure–property relationship and practical guidelines for optimizing injection-molded fiber-reinforced thermoplastic composites, particularly for thin-walled applications. The overall analytical framework of this study is illustrated in Fig 1.

## 2. Experimental methods and materials

### 2.1. Model information

**Fig 2:**
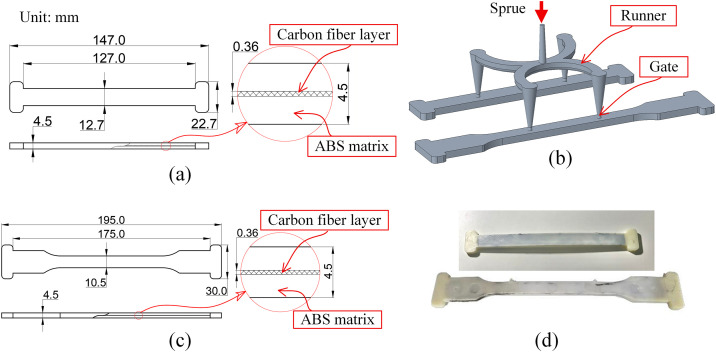
Geometry and molding system of ABS/carbon fiber composites specimens: (a) bending test specimen, (b) sprue–runner–gate system of the injection mold, (c) tensile test specimen, and (d) injection-molded sandwich-type composites parts.

In this study, two types of mechanical test specimens, namely bending specimens and tensile specimens, were designed to evaluate the mechanical properties of hybrid ABS/carbon fiber composites fabricated by injection molding. The geometry, material structure, and molding system of the specimens are illustrated in Fig 2. The bending specimen was designed in a straight bar configuration with an overall length of 147 mm and a gauge length of 127 mm Fig 2(a). The total thickness of the specimen was 4.5 mm. The tensile specimen had an overall length of 195 mm and a gauge length of 175 mm Fig 2(c). The width of the narrow section was 10.5 mm, and the total thickness was 4.5 mm, consisting of two main components: an ABS matrix layer occupying most of the cross-section and a carbon fiber layer positioned at the mid-plane along the thickness direction with an approximate thickness of 0.36 mm. This multilayer configuration forms a sandwich-type composites, in which the carbon fiber layer acts as the primary stiffness-enhancing and stress-transfer phase, while the ABS matrix provides bonding, load transfer, and processability for injection molding. The placement of the carbon fiber layer near the mid-plane of the cross-section is intended to improve structural symmetry and facilitate uniform resin impregnation, thereby reducing fiber misalignment and minimizing the risk of interlaminar delamination during the injection molding process.

The mold flow system was designed according to a standard sprue–runner–gate configuration, as shown in Fig 2(b). Molten ABS was introduced into the mold through a central sprue and subsequently distributed uniformly via the runner system to the gates leading into the specimen cavities. The gate locations were carefully arranged to ensure uniform melt flow and complete cavity filling, to enhance resin infiltration into the carbon fiber layer, and to minimize defects such as short shots, weld lines, and air entrapment. This flow system plays a critical role in promoting interfacial bonding between the ABS matrix and the carbon fiber layer, thereby directly influencing the mechanical performance and dimensional stability of the molded products after solidification. Fig 2d presents photographs of the fabricated ABS/CF composites specimens after the injection molding process. The bending and tensile specimens exhibit well-defined geometries, with the carbon fiber layer fully encapsulated within the ABS matrix. The specimen surfaces appear relatively uniform, and no major defects such as voids or surface cracking are observed. These results indicate that the specimen geometry design and mold flow system were suitable for producing sandwich-type composites materials using the insert injection molding process.

### 2.2. Material information

In this study, the polymer matrix material was ABS, specifically POLYLAC ABS PA-709S supplied by CHIMEI Corporation (Taiwan). The material was provided in the form of virgin pellets with a natural white color and was suitable for injection molding processes. ABS PA-709S was selected due to its balanced mechanical properties (Table 1), including good tensile strength and impact resistance, stable processability, and appropriate interfacial adhesion when combined with carbon fiber fabric reinforcement in insert injection molding experiments. The reinforcing material used was a woven carbon fiber fabric with a Plain Weave 3K architecture and an areal density of 240 g/m², supplied by Tchaintech. This carbon fabric consists of 3K carbon fiber tows woven in a plain weave pattern, providing a favorable balance between strength, elastic modulus, and resin permeability during the insert injection molding process. The characteristics of the carbon fiber fabric, including its areal weight, fiber arrangement, and fabric dimensions, directly influence the mechanical performance, residual stress distribution, and interfacial bonding efficiency between the ABS matrix and the reinforcing fibers (Table 2).

**Table 1:** Material properties of the investigated ABS polymer.

Commercial product name	POLYLAC ABS PA-709S
Solid density (g/cm3)	1.04 g/cm³
Material structure	Amorphous terpolymer (ABS)
Melt flow rate	22 g/10 min
Thermal conductivity (W/m°C)	0.17 W/m·°C
Tensile elastic modulus, 1 mm/min (MPa)	2100 MPa
Tensile strength	45 MPa
Poisson ratio	0.38
Mold shrinkage	0.4–0.8%
Ejection temperature (°C)	170–320 °C

**Table 2:** Material properties of the investigated carbon fiber (CF).

Material property	Value
Fiber type	Carbon Fiber 3K
Weave pattern	Plain weave
Areal density (g/m²)	240
Fabric thickness (mm)	0.36
Warp/weft ratio	1:1
Fiber composition	100% carbon
Single-fiber tensile strength	4000 Mpa [28]
Fiber elastic modulus	230 GPa

### 2.3. The injection mold

**Fig 3:**
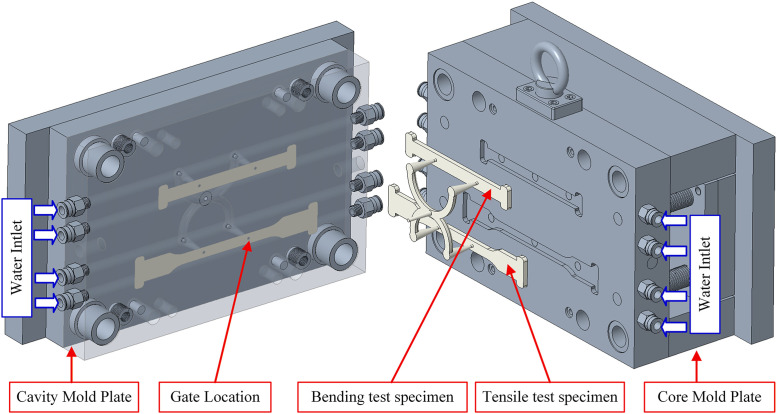
Injection mold configuration illustrating the cavity-side and core-side mold plates, water inlets, gate location, specimen bending deformation, and tensile test specimen used for mechanical evaluation.

The injection mold system was specifically designed and fabricated to produce hybrid ABS/carbon fiber composite specimens using the insert injection molding process. The overall mold configuration is shown in Fig 3. and consists of two main mold halves: the cavity mold plate and the core mold plate. These components were assembled coaxially on the injection molding machine to ensure geometric accuracy and tight sealing throughout the molding cycle. On each mold half, the cooling water channel system (water inlets) was symmetrically arranged and distributed along the molding region, enabling uniform and stable temperature control during the injection, packing, and cooling stages. The use of multiple water inlets enhances heat transfer efficiency and minimizes temperature gradients within the mold cavity, thereby reducing thermal deformation, residual stresses, and dimensional deviations of the molded parts after solidification. The melt delivery system was designed according to a sprue–runner–gate configuration, in which the gate location was optimally positioned at the center of the distribution system to ensure symmetric and uniform melt flow into the mold cavities. This configuration allows the molten ABS to effectively encapsulate the pre-inserted carbon fiber fabric layer while minimizing flow stratification, flow instability, and surface defects at the material interface region.

**Fig 4:**
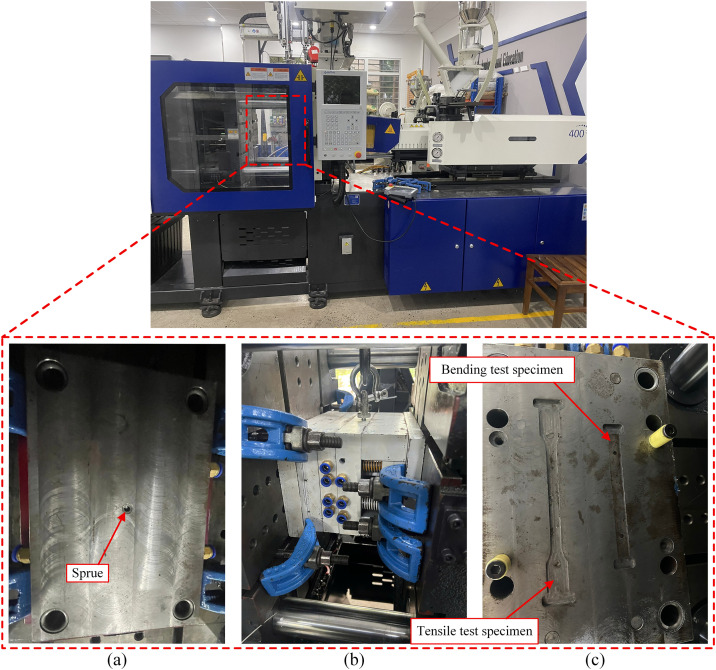
Injection molding setup and mold cavity configuration: (a) sprue location of the mold, (b) Haitian MA1200 III injection molding machine during operation, and (c) mold cavities for bending test specimen and tensile test specimen.

The actual mold configuration and operating process are illustrated in Fig 4. The sprue was positioned at the center of the mold Fig 4(a) to ensure a symmetric pressure distribution as the molten polymer entered the runner system. The mold assembly was installed and operated on a Haitian MA1200 III injection molding machine Fig 4(b), which enabled precise control of key processing parameters, including injection pressure, packing pressure, melt temperature, mold temperature, and cycle time. Fig 4(c) presents the two mold cavities designed for the bending and tensile test specimens. The cavity geometry was manufactured with high precision to ensure uniform specimen thickness, geometric accuracy, and high repeatability across molding cycles. This level of dimensional consistency is particularly critical for process parameter optimization studies, as geometric deviations and variations in molding conditions can introduce significant noise into mechanical property measurements and subsequent statistical analyses.

**Fig 5:**
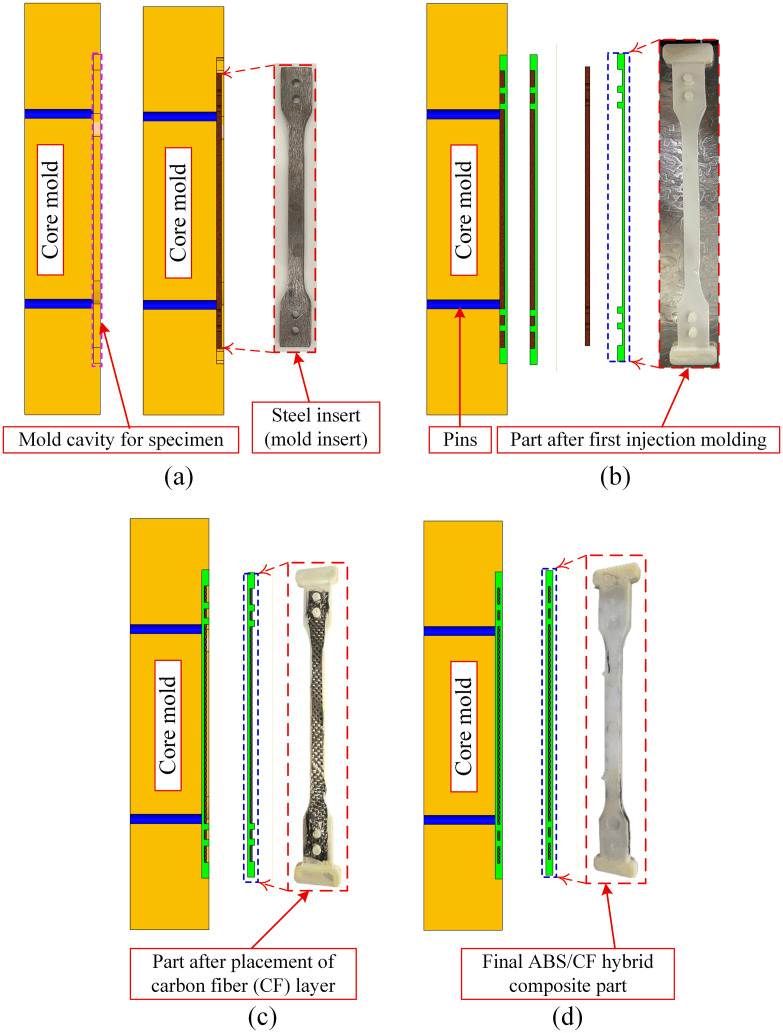
Schematic illustration of the two-step injection molding process for fabricating ABS/carbon fiber (CF) sandwich-type composites specimens: (a) mold cavity configuration with steel insert for the first injection molding step, (b) preformed ABS part after the first injection molding and removal of the steel insert, (c) part after placement of the carbon fiber (CF) fabric layer into the mold cavity, and (d) final ABS/CF composites part after the second injection molding step.

A Haitian MA 1200 III injection molding machine (clamping force: 1200 kN; screw diameter: 35 mm; maximum injection pressure: 243 MPa; injection volume: 134 cm³) was used to inject molten ABS into the mold cavity at predefined speeds and pressures. Maintaining a high mold surface temperature facilitated smooth melt flow and effective penetration through the pre-positioned carbon fiber (CF) fabric layer, thereby reducing common defects such as weld lines and short shots. The fabrication of hybrid ABS/carbon fiber (CF) composite specimens was carried out using a two-step injection molding process to ensure accurate positioning of the carbon fabric layer and to enhance interfacial adhesion between the ABS matrix and the fiber reinforcement. The schematic of the entire manufacturing procedure is illustrated in Fig 5.

In the first injection step Fig 5(a, b), the mold cavity was equipped with a steel insert positioned at the center of the cavity to preform the ABS matrix. This steel insert acted as a temporary core to create a controlled hollow region within the molded part. During this stage, molten ABS was injected into the cavity through the runner and gate system, completely filling the mold volume surrounding the steel insert. After completion of the filling, packing, and cooling stages, an intermediate product was obtained in the form of a preformed ABS part with stable geometry and an internal void corresponding to the shape of the steel insert. The insert was subsequently removed, leaving a cavity with defined dimensions and geometry suitable for placement of the carbon fiber reinforcement layer in the subsequent molding step Fig 5(b).

In the second injection step Fig 5(c, d), the carbon fiber fabric layer was precisely placed into the cavity of the preformed ABS part. The positioning of the CF layer was assisted by locating pins to ensure accurate and uniform alignment and to prevent displacement of the reinforcement layer during mold closure. After the CF layer was fixed in position, the mold was closed and molten ABS was injected again to encapsulate and impregnate the surface of the carbon fabric. During this stage, the molten polymer penetrated into the interstitial spaces between the fiber bundles of the woven carbon fabric, forming both mechanical interlocking and thermal bonding between the polymer matrix and the reinforcement layer. The subsequent packing and cooling stages stabilized the composite structure and minimized the formation of air voids and interfacial defects.

The final product obtained was a hybrid ABS/CF composite specimen Fig 5(d), in which the carbon fiber fabric layer was fully integrated within the ABS matrix, forming a layered sandwich-type structure. Compared with neat ABS specimens, this structure exhibits enhanced tensile and flexural performance. This improvement is attributed not only to the reinforcing effect of the carbon fiber layer but also to improved interfacial bonding and structural integrity of the composite.

The two-step injection molding approach enables precise control over the reinforcement layer position, ensures high geometric repeatability, and significantly improves the overall mechanical performance of the composite material. In addition, this process is compatible with conventional injection molding technology and does not require major modifications to the molding equipment, indicating strong potential for large-scale production of complex-shaped composites components.

### 2.4. Tensile and flexural testing equipment

#### 2.4.1. The tensile test.

**Fig 6:**
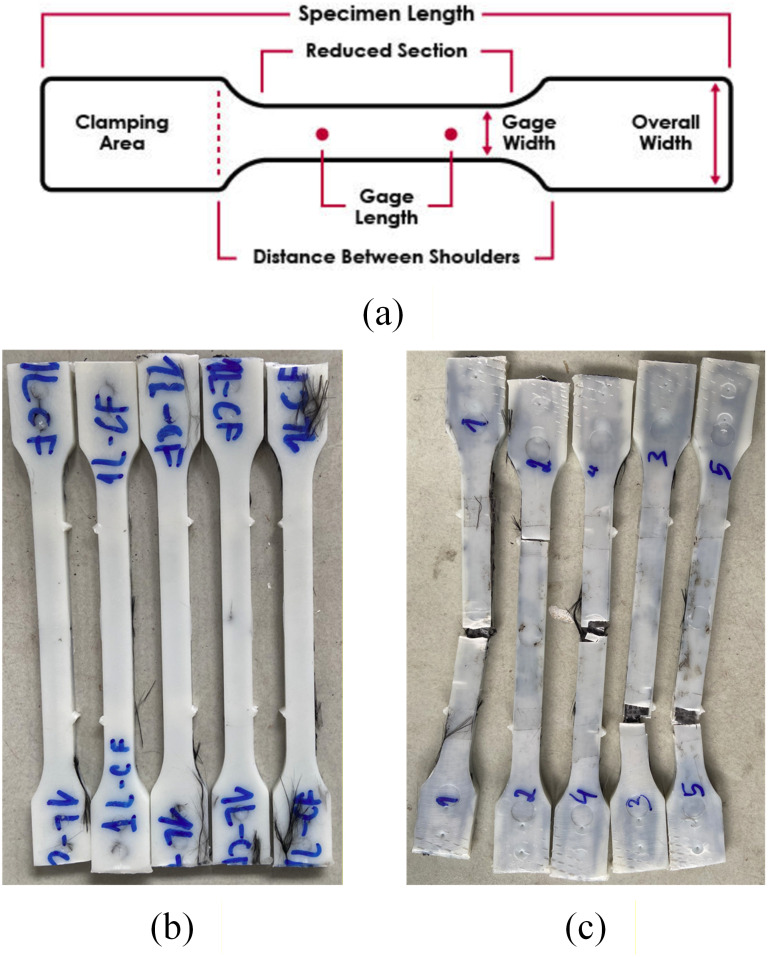
Tensile test of ABS/CF composite specimens according to ASTM D638: (a) Geometry of a standard ASTM D638 dog-bone tensile specimen [29]. (b) specimens before testing; (c) fractured specimens after tensile testing.

Tensile tests were conducted to determine the fundamental mechanical properties of the materials, including tensile strength, yield stress, elongation at break, and tensile modulus. All tests were performed in accordance with the relevant ASTM standards for polymers and fiber-reinforced composite materials. For both neat ABS and ABS/CF composites, tensile specimens were fabricated in the dog-bone geometry (Fig 6) following the ASTM D638 Type I standard for plastics and polymer matrix composites [29]. This specific standard is frequently utilized in the mechanical characterization of advanced hybrid structures, such as in machine learning-based predictive models for fiber-reinforced composites [30]. The specimens were clamped into the grips of a universal testing machine and subjected to uniaxial tensile loading until complete fracture occurred.

The tensile specimens had overall dimensions of L=175 mm, W=20 mm, and t=4.5 mm. In the gauge section, the thickness was maintained at 4.5 mm and the width was 10.5 mm, while the fillet radius and gauge length were controlled in strict accordance with the standard specifications. The specimens were clamped into the grips of a universal testing machine and subjected to uniaxial tensile loading until complete fracture occurred.

The tensile testing speed was selected in accordance with the low strain rate requirements specified by ASTM standards, corresponding to a crosshead displacement rate in the range of 5–50 mm/min depending on the material type. In this study, a tensile speed of v=5 mm/min was applied.

During testing, the tensile load P (N) and the elongation ΔL (mm) were continuously recorded. The nominal tensile stress was calculated as:


$$\begin{array}{c} \sigma_{\text{t}}=\;\frac{\text{P}}{{\text{A}}_{\text{0}}}\; \end{array}$$ (1)

Where $\sigma_{t}$ is the tensile stress (MPa), P is the instantaneous tensile load (N), and $A_{\text{0}}$ is the original cross-sectional area of the specimen (mm²).

The axial tensile strain was determined by:


$$\begin{array}{c} \epsilon_{\text{t}}=\;\frac{\Delta \text{L}}{{\text{L}}_{\text{0}}} \end{array}$$ (2)

Where ΔL is the specimen elongation (mm), and $L_{\text{0}}$ is the gauge length specified by the standard (mm).

The tensile modulus was obtained from the initial linear region of the stress–strain curve. The ultimate tensile strength was identified as the maximum stress value immediately prior to specimen fracture. For ABS/CF composite materials, distinct failure modes such as interlaminar delamination, fiber breakage, and mixed brittle–ductile fracture behavior were clearly observed in the gauge section.

#### 2.4.2. Flexural testing equipment.

**Fig 7:**
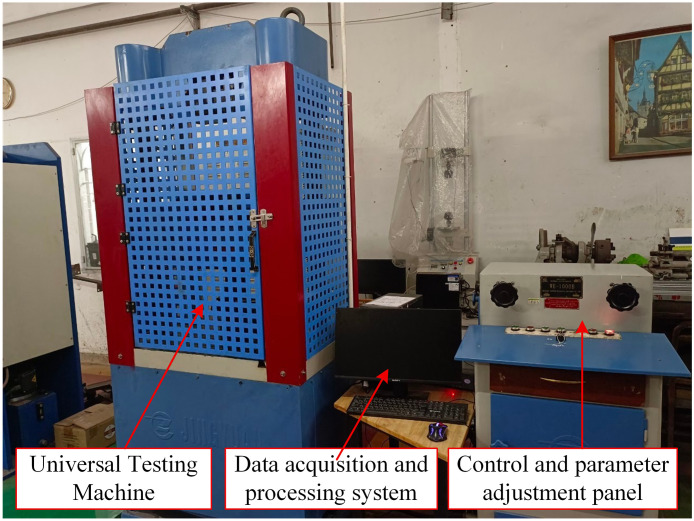
Experimental setup of the universal testing machine (JingYuan WE-1000B).

Bending and tensile tests were conducted using a JingYuan WE-1000B universal testing machine (Fig 7) with a maximum load capacity of 1000 kN. For bending tests, a three-point bending fixture was employed. The specimens were placed on two parallel supports, and the load was applied at the mid-span of the specimen in a direction perpendicular to its longitudinal axis. The tests were performed at a constant crosshead displacement rate of 2 mm/min until specimen failure or until the maximum load was reached. The bending load and mid-span deflection were recorded to calculate the maximum bending stress.

For tensile tests, the specimens were firmly clamped at both ends and subjected to uniaxial tensile loading at a constant crosshead speed of 2 mm/min until complete fracture occurred. During testing, force–displacement data were continuously acquired and converted into stress–strain curves to determine the maximum tensile stress. All experiments were carried out under ambient laboratory conditions at a temperature of 28 °C and a relative humidity of approximately 70%. For each experimental condition, three replicate specimens were tested to ensure data reliability and to account for experimental variability.

### 2.5. Taguchi grey correlation analysis

The TGC method is one of the most widely used robust multi-objective optimization approaches for process parameter optimization [19]. This method is capable of improving the quality of multiple performance characteristics simultaneously with high accuracy [20,21]. Therefore, multi-objective optimization in this study was performed using the TGC approach. According to the optimization objective of this work, the optimization problem of injection molding process parameters can be treated as a static problem with a larger-the-better S/N ratio, which is expressed as follows:


$$\begin{array}{c} {\text{N}}_{\text{s}}=\;-\text{10log}\left(\frac{\text{1}}{\text{n}}\sum_{\text{i}=\text{1}}^{\text{n}}\frac{\text{1}}{\overset{\text{2}}{\underset{\text{i}}{\text{y}}}}\right) \end{array}$$ (3)

where $N_{s}$ is the S/N ratio, $y_{i}$ represents the measured response value at the i-th repetition, and nis the number of samples in each experimental run. This formulation promotes an increase in the mean response value while simultaneously reducing its variability, thereby enhancing process stability.

The S/N ratios of each individual performance characteristic were normalized to the range of [0, 1] using the grey relational normalization equation, as given in Eq. (4). The normalized data were then used to calculate the grey relational coefficients according to Eq. (5). Subsequently, the TGC grade was determined by averaging the grey relational coefficients using Eq. (6) [21].


$$\begin{array}{c} \overset{*}{\underset{i}{X}}(k)=\;\frac{\text{max}\overset{\left(\text{0}\right)}{\underset{i}{X}}(k)-\;\overset{\left(\text{0}\right)}{\underset{i}{X}}(k)}{\text{max}\overset{\left(\text{0}\right)}{\underset{i}{X}}(k)-min\;\overset{\left(\text{0}\right)}{\underset{i}{X}}(k)} \end{array}$$ (4)

where $\overset{*}{\underset{i}{X}}(k)$ denotes the normalized S/N ratio sequence, and $\text{max}\overset{\left(\text{0}\right)}{\underset{i}{X}}(k)$ and $\text{min}\overset{\left(\text{0}\right)}{\underset{i}{X}}(k)$ represent the maximum and minimum values of the original sequence $\overset{\left(\text{0}\right)}{\underset{i}{X}}(k)$, respectively.


$$\begin{array}{c} \varepsilon \left(X_{i}(k),X_{j}(k)\right)=\;\frac{\Delta_{min}+\;\xi \Delta_{max}}{\Delta_{\text{0i}}(k)+\;\xi \Delta_{max}} \end{array}$$ (5)

where $\varepsilon (X_{i}(k),X_{j}(k))$is the grey relational coefficient, $\Delta_{\text{0}i}(k)$is the absolute difference between the reference sequence $X_{\text{0}}(k)$and the comparability sequence $X_{i}(k)$, and ξis the distinguishing coefficient, which is commonly set to 0.5.


$$\begin{array}{c} R\left(X_{i},X_{j}\right)=\;\frac{\text{1}}{n}\sum_{k=\text{1}}^{n}\varepsilon \left(X_{i}(k),X_{j}(k)\right) \end{array}$$ (6)

Based on the selected processing parameters, the experimental design in this study was developed using a systematic and scientific approach to comprehensively evaluate the effects of injection molding parameters on the mechanical properties of hybrid ABS/carbon fiber composites. Five principal processing parameters—namely filling pressure (A), packing pressure (B), filling time (C), packing time (D), and melt temperature (E)—were identified as the most critical control variables governing mold filling behavior, resin impregnation into the carbon fiber fabric layer, interfacial bonding between fiber and matrix, and the resulting microstructure formed during solidification. The selection of these parameters is rooted in the rheological behavior of molten polymers and the stress transfer mechanisms in composite materials. In particular, the filling and packing stages play a decisive role in determining material densification, the formation of internal defects (such as voids, weld lines, and short shots), and the overall mechanical homogeneity of the molded parts.

Specifically, the injection pressure was varied from 104 to 118 MPa to ensure that the molten polymer possessed sufficient kinetic energy to completely fill the mold cavity containing the Plain Weave 3K carbon fiber fabric layer, while preventing premature solidification in thin-walled regions. An excessively low injection pressure may result in incomplete filling defects, whereas an excessively high pressure can cause fiber displacement or induce residual stresses in the molded parts. The packing pressure, ranging from 96 to 100 MPa, was applied during the packing stage to compensate for volumetric shrinkage of the polymer during the phase transition from melt to solid, thereby improving material densification and reducing internal void formation.

Meanwhile, the filling time (1–3 s) directly influences the melt flow rate and shear conditions of the polymer, which in turn affect molecular orientation and resin distribution around the carbon fiber layer. The packing time (0.5–2.5 s) determines the duration over which pressure is maintained during the initial solidification stage and plays a critical role in stabilizing geometric dimensions and enhancing the mechanical performance of the molded products. Finally, the melt temperature, controlled within the range of 228–232 °C, governs the viscosity of ABS, the wetting behavior of the carbon fibers, and the interfacial bonding strength between the matrix and reinforcement phases. Inappropriate melt temperatures may lead to polymer degradation or deterioration of mechanical properties.

To simultaneously investigate the effects of five processing parameters at five different levels, the Taguchi orthogonal experimental design (OED) was employed using an L25 (5⁵) array. The selection of the L25 orthogonal design significantly reduced the required number of experiments from 3125 combinations in a full factorial design to only 25 representative trials, while still preserving statistical independence among factors and enabling the individual contribution of each parameter to the mechanical responses to be evaluated. The levels of each factor were uniformly distributed within the investigated ranges, as summarized in Table 3, in order to capture the continuous variation trends of mechanical properties with respect to processing conditions.

**Table 3:** Factors and levels of the test.

Levels	Factors				
	Filling pressure (MPa)	Packing pressure (MPa)	Filling time (s)	Packing time (s)	Melt temperature (°C)
**1**	104	96	1	0.5	228
**2**	106	97	1.5	1	229
**3**	110	98	2	1.5	230
**4**	114	99	2.5	2	231
**5**	118	100	3	2.5	232

The orthogonal experimental design matrix (Table 4) specifies the detailed combinations of injection molding parameters used to fabricate the bending and tensile specimens. Two performance indices, namely bending deformation (R1) and tensile deformation (R2), were defined as the desired “larger-the-better” responses. The values of R1 and R2 for each experimental run were obtained from mechanical testing of the injection-molded specimens produced under the corresponding parameter settings. The results of the orthogonal experimental design are presented in Tables 5 and 6.

**Table 4:** Orthogonal experimental design table.

Number	Filling pressure (MPa)	Packing pressure (MPa)	Filling time (s)	Packing time (s)	Melt temperature (°C)
**1**	104	96	1	0.5	228
**2**	104	97	1.5	1	229
**3**	104	98	2	1.5	230
**4**	104	99	2.5	2	231
**5**	104	100	3	2.5	232
**6**	106	96	1.5	1.5	231
**7**	106	97	2	2	232
**8**	106	98	2.5	2.5	228
**9**	106	99	3	0.5	229
**10**	106	100	1	1	230
**11**	110	96	2	2.5	229
**12**	110	97	2.5	0.5	230
**13**	110	98	3	1	231
**14**	110	99	1	1.5	232
**15**	110	100	1.5	2	228
**16**	114	96	2.5	1	232
**17**	114	97	3	1.5	228
**18**	114	98	1	2	229
**19**	114	99	1.5	2.5	230
**20**	114	100	2	0.5	231
**21**	118	96	3	2	230
**22**	118	97	1	2.5	231
**23**	118	98	1.5	0.5	232
**24**	118	99	2	1	228
**25**	118	100	2.5	1.5	229

**Table 5:** Flexural and tensile strength of neat ABS (mean ± standard deviation; n = 3).

Number	Flexural strength (MPa)	S/N R_A1_	Tensile strength (MPa)	S/N R_A2_
**1**	5.86 ± 0.01	15.3530	33.90 ± 0.06	30.6031
**2**	6.24 ± 0.02	15.8991	34.33 ± 0.11	30.7126
**3**	6.05 ± 0.01	15.6399	33.41 ± 0.11	30.4767
**4**	6.25 ± 0.01	15.9130	33.71 ± 0.15	30.5543
**5**	6.09 ± 0.03	15.6923	33.87 ± 0.09	30.5954
**6**	6.21 ± 0.01	15.8572	34.07 ± 0.08	30.6474
**7**	6.38 ± 0.01	16.0919	34.57 ± 0.13	30.7740
**8**	6.21 ± 0.03	15.8618	33.35 ± 0.22	30.4628
**9**	5.98 ± 0.02	15.5340	33.70 ± 0.10	30.5526
**10**	6.08 ± 0.01	15.6781	33.39 ± 0.15	30.4732
**11**	6.25 ± 0.01	15.9176	33.56 ± 0.13	30.5156
**12**	5.93 ± 0.02	15.4562	33.91 ± 0.07	30.6066
**13**	6.13 ± 0.03	15.7445	34.56 ± 0.22	30.7706
**14**	6.20 ± 0.08	15.8432	34.01 ± 0.10	30.6330
**15**	6.00 ± 0.03	15.5630	34.15 ± 0.24	30.6670
**16**	6.16 ± 0.03	15.7963	34.07 ± 0.07	30.6474
**17**	6.06 ± 0.02	15.6542	33.93 ± 0.16	30.6108
**18**	6.08 ± 0.01	15.6828	33.98 ± 0.09	30.6253
**19**	6.12 ± 0.02	15.7303	34.38 ± 0.10	30.7270
**20**	6.13 ± 0.02	15.7539	34.81 ± 0.11	30.8341
**21**	6.19 ± 0.07	15.8291	35.07 ± 0.24	30.8987
**22**	6.13 ± 0.06	15.7539	34.51 ± 0.24	30.7589
**23**	6.49 ± 0.01	16.2449	33.93 ± 0.31	30.6108
**24**	6.36 ± 0.03	16.0646	34.85 ± 0.12	30.8432
**25**	6.21 ± 0.03	15.8618	35.17 ± 0.10	30.9243

S/N ratio (R_A1_: flexural strength; R_A2_: tensile strength, larger-is-better criterion).

**Table 6:** Flexural and tensile strength of carbon fiber-reinforced ABS (ABS/CF) composite (mean ± standard deviation; n = 3).

Number	Flexural strength (MPa)	S/N R_A1_	Tensile strength (MPa)	S/N R_A2_
**1**	5.93 ± 0.01	15.4660	38.59 ± 0.19	31.7295
**2**	6.30 ± 0.02	15.9868	38.94 ± 0.11	31.8070
**3**	5.79 ± 0.01	15.2586	39.15 ± 0.14	31.8546
**4**	6.61 ± 0.03	16.4084	39.60 ± 0.22	31.9539
**5**	6.46 ± 0.03	16.2002	38.89 ± 0.16	31.7975
**6**	6.04 ± 0.02	15.6207	39.23 ± 0.17	31.8731
**7**	6.56 ± 0.05	16.3381	39.50 ± 0.13	31.9327
**8**	6.45 ± 0.02	16.1957	38.20 ± 0.28	31.6420
**9**	6.40 ± 0.02	16.1191	39.01 ± 0.08	31.8235
**10**	6.58 ± 0.01	16.3601	38.67 ± 0.21	31.7482
**11**	5.76 ± 0.03	15.2034	38.73 ± 0.19	31.7617
**12**	6.59 ± 0.01	16.3733	38.89 ± 0.17	31.7968
**13**	6.68 ± 0.05	16.4955	39.13 ± 0.18	31.8502
**14**	6.77 ± 0.05	16.6160	39.00 ± 0.12	31.8205
**15**	6.68 ± 0.04	16.4999	38.74 ± 0.24	31.7639
**16**	6.72 ± 0.02	16.5517	38.99 ± 0.15	31.8183
**17**	6.71 ± 0.02	16.5388	38.56 ± 0.22	31.7235
**18**	6.59 ± 0.03	16.3733	38.24 ± 0.18	31.6511
**19**	6.68 ± 0.03	16.4999	39.85 ± 0.14	32.0086
**20**	6.65 ± 0.01	16.4521	40.40 ± 0.22	32.1283
**21**	6.82 ± 0.01	16.6757	39.57 ± 0.22	31.9473
**22**	6.67 ± 0.03	16.4869	40.04 ± 0.19	32.0506
**23**	6.93 ± 0.01	16.8105	39.93 ± 0.13	32.0253
**24**	6.69 ± 0.02	16.5128	40.16 ± 0.19	32.0766
**25**	6.67 ± 0.01	16.4825	41.27 ± 0.14	32.3120

S/N ratio (R_A1_: flexural strength; R_A2_: tensile strength, larger-is-better criterion).

Through this approach, the relationship between injection molding processing conditions and the quality indicators (bending strength and tensile strength) of ABS/CF composite materials can be quantitatively and systematically analyzed. The experimental data obtained from the L25 orthogonal array serve as the basis for applying statistical analysis methods such as S/N ratio analysis, ANOVA, and grey relational analysis (GRA). These methods enable the determination of the relative influence of each processing parameter as well as the identification of the optimal parameter combination for sandwich-type composites injection molding.

This integrated methodology not only facilitates the optimization of mechanical properties of the molded products but also contributes to establishing a scientific framework for the design of injection molding processes for polymer matrix composites reinforced with woven fibers in engineering applications that require high mechanical strength and good dimensional stability.

### 2.6. The bending test

**Fig 8:**
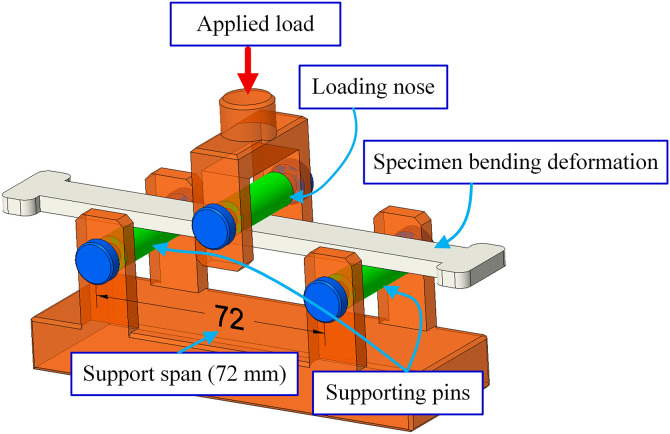
Schematic illustration of the three-point bending test setup, showing the applied load, loading nose, supporting pins, support span (72 mm), and bending deformation of the specimen.

After the fabrication of the test specimens, bending tests were conducted to evaluate the flexural strength and bending response of the materials under applied loading. In this test, each specimen was supported at two points and subjected to a load applied at the midpoint or at a specified location to generate a bending moment Fig 8. According to the ASTM D790 standard, the support span for specimens with a thickness of 4.5 mm is determined using the relation L=16d, where d is the specimen thickness; therefore, the span length between the two supports was set to 72 mm. This standard has been successfully applied in recent literature to evaluate the mechanical performance of specialized composites, such as those with reinforced weave patterns [31] and epoxy-cotton seed particle structures [24]. In addition, the specimen width (W) and thickness (T) were measured prior to testing, as required by the standard [32,33].

The crosshead displacement rate was calculated in accordance with ASTM recommendations based on a strain rate of 0.01 mm/mm/min. The bending speed was determined using the following equation:


$$\begin{array}{c} \text{R}=\;\frac{{\text{ZxL}}^{\text{2}}}{\text{6xd}} \end{array}$$ (7)

Where R is the crosshead displacement rate (mm/min), L(or S) is the support span of the specimen (mm), d(or T) is the specimen thickness (mm), and Z=0.01 mm/mm/min is the recommended strain rate according to ASTM D790.

For the bending strength test in this study, the displacement rate was calculated as follows:


$$\begin{array}{c} \text{R}=\;\frac{{\text{ZxL}}^{\text{2}}}{\text{6xd}}=\frac{{\text{0},\text{01x72}}^{\text{2}}}{\text{6x4},\text{5}}=\text{3}.\text{20}\times {\text{1}\text{0}}^{-\text{5}}\text{ m}/\text{s} \end{array}$$ (8)

However, the minimum operating crosshead speed of the bending testing machine was $R_{\text{min}}=\text{15}$ mm/min. Therefore, this speed was selected for all bending tests.

After completion of the bending tests, the output data consisted of the applied load P(N) and displacement D(mm). These data were subsequently used to construct stress–strain curves to describe the relationship between stress and strain of the bending specimens. For each experimental condition, one representative specimen was selected to generate the corresponding stress–strain curve.

According to the ASTM D790 standard for flexural testing, the flexural stress and flexural strain were calculated using the following equations.

The flexural stress was determined as:


$$\begin{array}{c} \sigma_{\text{f}}=\;\frac{\text{3PL}}{\text{2b}{\text{d}}^{\text{2}}} \end{array}$$ (9)

Where $\sigma_{f}$ is the flexural stress (MPa), P is the applied load (N), L (or S) is the support span, which was fixed at 72 mm, b(or w) is the specimen width (mm), and d (or T) is the specimen thickness (mm).

The flexural strain was calculated as:


$$\begin{array}{c} \epsilon_{\text{f}}=\;\frac{\text{6Dd}}{{\text{L}}^{\text{2}}} \end{array}$$ (10)

Where $\varepsilon_{f}$ is the flexural strain (mm/mm), and Dis the mid-span deflection of the specimen under load (mm).

**Fig 9:**
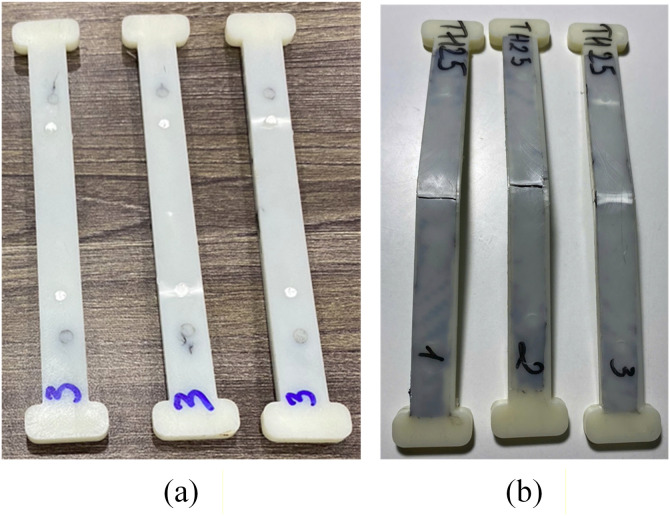
Three‐point bending test of ABS/CF composite specimens: (a) molded specimens before testing, and (b) specimens after bending failure.

The relationship between flexural stress and flexural strain was used to construct stress–strain curves, which were employed to evaluate the flexural stiffness and failure behavior of the carbon fiber–reinforced composite materials Fig 9.

## 3. Results and discussion

### 3.1. Taguchi–ANOVA analysis of bending and tensile test results

**Fig 10:**
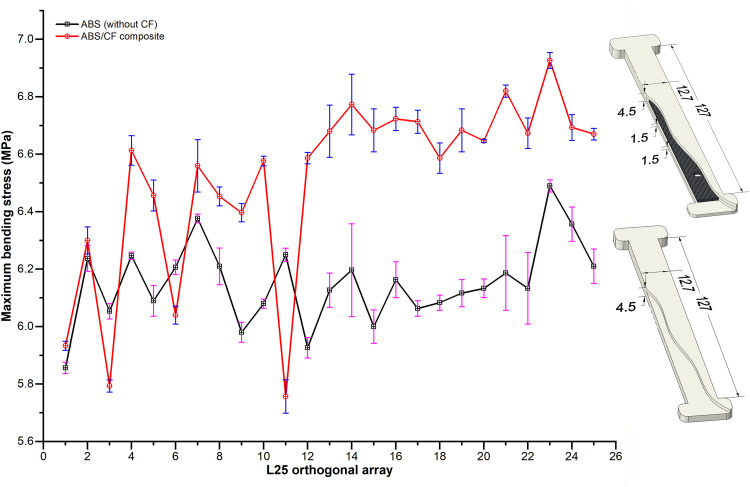
Maximum bending stress of ABS and ABS/CF composite under L25 orthogonal design.

**Fig 11:**
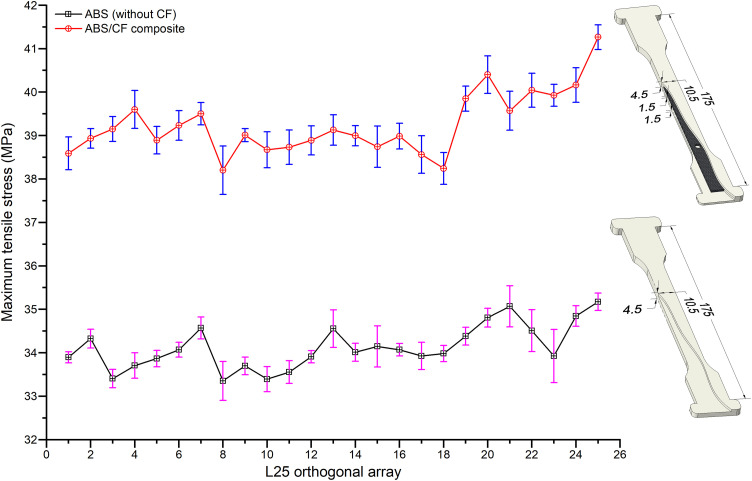
Maximum tensile stress of ABS and ABS/CF composite under L25 orthogonal design.

The experimental results of bending and tensile strength tests are summarized in Table 5 (neat ABS), Table 6 (ABS/CF composite), and the corresponding plots in Fig 10 and 11. These results clearly demonstrate that the incorporation of the carbon fiber (CF) layer significantly enhances both the maximum flexural stress and the maximum tensile stress of ABS under the same Taguchi L25 orthogonal design.

For the bending tests, neat ABS exhibited maximum flexural stress values ranging from 5.86 to 6.49 MPa, with the highest value of 6.49 MPa obtained in experiment No. 23. In contrast, the ABS/CF composite showed a substantially higher range, from 5.76 to 6.93 MPa, with a maximum value of 6.93 MPa also recorded in experiment No. 23. Accordingly, the maximum flexural strength increased by approximately 6.8–7.0% compared with neat ABS.

This improvement reflects the dominant reinforcing role of the carbon fiber fabric layer in the tensile region under bending conditions, where it contributes to stress redistribution and suppresses the initiation and propagation of microcracks within the ABS matrix. This reinforcement mechanism is consistent with previous studies, in which carbon fibers act as a high-modulus reinforcing phase, thereby enhancing the stiffness and flexural resistance of thermoplastic composites [5].

A similar trend was observed in the tensile tests. According to Table 5, the maximum tensile stress of neat ABS ranged from 33.35 to 35.17 MPa, with the highest value of 35.17 MPa obtained in experiment No. 25. In contrast, the ABS/CF composite in Table 6 exhibited markedly higher values, varying from 38.20 to 41.27 MPa, with a maximum tensile stress of 41.27 MPa also recorded in experiment No. 25. This corresponds to an increase in tensile strength of approximately 17–18%, indicating the pronounced effectiveness of the CF layer in transferring applied stress from the polymer matrix to the fiber phase through interfacial bonding.

Furthermore, the plots in Figs 10 and 11 show that the response curves of the ABS/CF composite not only have higher mean values but also display improved stability compared with neat ABS. This suggests that the hybrid ABS/CF structure reduces the sensitivity of mechanical properties to variations in processing parameters such as filling pressure, packing pressure, filling time, and melt temperature.

Overall, the two material systems demonstrate that the ABS/CF composite consistently outperforms neat ABS in both flexural and tensile performance across all 25 combinations of processing parameters. These findings are in good agreement with previous studies, which reported that carbon fiber–reinforced thermoplastic composites exhibit significant improvements in mechanical strength due to the stress transfer capability of the fibers and enhanced interfacial interactions between the fiber and polymer matrix [5].

Moreover, the present results are consistent with investigations on multilayer composite injection molding, where optimization of processing parameters was shown to enhance fiber wetting, reduce internal defects, and consequently improve the mechanical properties of the final products [32]. Therefore, the integration of a carbon fiber layer within a two-step injection molding process represents an effective approach to substantially enhance the mechanical performance of ABS materials, offering strong potential for applications requiring high strength and stiffness, such as precision mechanical components and automotive engineering industries.

The results of the analysis of variance (ANOVA) for the mechanical responses are presented in Table 7, where the p-values are used to evaluate the statistical significance of each processing parameter. In addition, the F-values and the coefficient of determination (R²), obtained from the general linear regression model, were calculated to assess the adequacy of the model and the relative influence of each factor on the responses. Specifically, for the ABS/CF composite under flexural testing, filling pressure and packing pressure exhibit statistically significant effects, with p-values of 0.012 and 0.039 (p < 0.05), respectively. These factors are also associated with higher F-values compared to the remaining parameters, indicating their dominant roles in controlling the mechanical performance of the composite. In contrast, for neat ABS, all p-values exceed 0.05, suggesting that there is insufficient statistical evidence to confirm the significance of the processing parameters within the investigated range.

**Table 7:** Comparative ANOVA results of injection molding parameters for ABS and ABS/CF composites.

Factor	Bending ABS (p)	Bending ABS/CF (p)	Tensile ABS (p)	Tensile ABS/CF (p)
Filling pressure	0.348	0.012*	0.388	0.072
Packing pressure	0.836	0.039*	0.866	0.344
Filling time	0.383	0.063	0.954	0.393
Packing time	0.722	0.100	0.917	0.834
Melt temperature	0.363	0.077	0.951	0.351

* *p < 0.05 indicates statistical significance*

Table 8 presents the percentage contribution of each factor based on the adjusted sum of squares (Adj SS), providing a quantitative assessment of the relative influence of the processing parameters. The results indicate that filling pressure is the most dominant factor across all four cases, particularly for the ABS/CF composite, with contributions reaching 38.8% for flexural strength and 47.08% for tensile strength. The consistency among the p-value analysis, F-value trends, and percentage contribution results confirms the reliability of the ANOVA findings. Furthermore, these results suggest that the incorporation of carbon fiber reinforcement significantly increases the sensitivity of mechanical properties to pressure-related parameters and melt flow conditions during the injection molding process.

**Table 8:** Percentage contribution of injection molding parameters to bending and tensile strength of ABS and ABS/CF composites based on ANOVA.

Factor	Bending ABS		Bending ABS/CF		Tensile ABS		Tensile ABS/CF	
**Adj SS**	**(%)**	**Adj SS**	**(%)**	**Adj SS**	**(%)**	**Adj SS**	**(%)**
Filling pressure	0.11460	24.39	0.91148	38.8	2.6605	42.70	5.8439	47.08
Packing pressure	0.02612	5.56	0.46559	19.8	0.5849	9.39	1.7619	14.19
Filling time	0.10367	22.06	0.34612	14.8	0.2943	4.72	1.5308	12.33
Packing time	0.04017	8.55	0.25652	10.9	0.4214	6.76	0.4004	3.23
Melt temperature	0.10986	23.38	0.30437	13.0	0.3044	4.89	1.7265	13.91

**(%)** *Percentage contribution calculated based on Adj SS / Total SS.*

### 3.2. S/N Ratio Analysis

**Fig 12:**
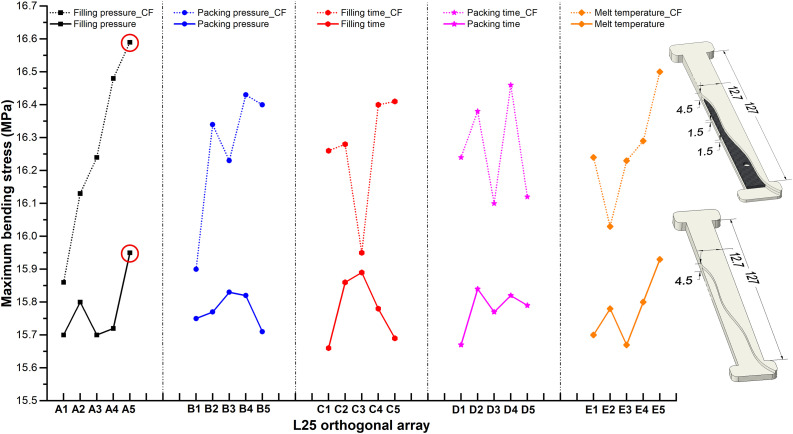
Signal-to-noise ratios of bending strength for ABS and ABS/CF composite.

**Fig 13:**
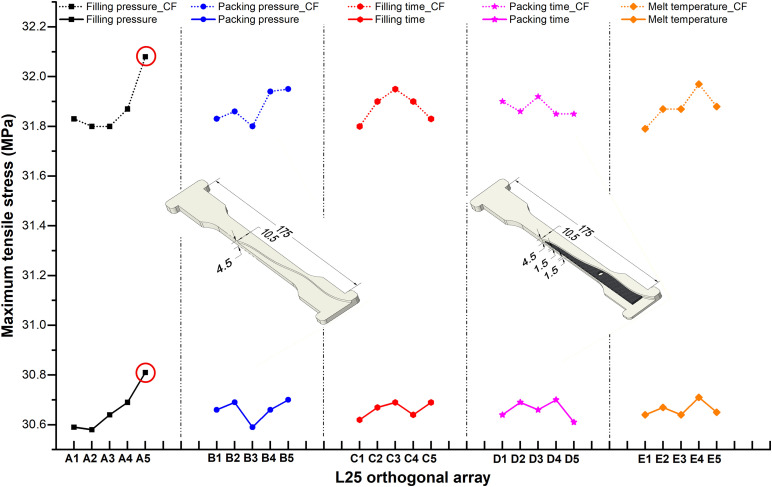
Signal-to-noise (S/N) ratios of tensile strength for ABS and ABS/CF composite under L25 orthogonal design.

The results of the S/N ratio analysis presented in Tables 9 and 12 and Figs 12 and 13 quantitatively evaluate the effects of injection molding processing parameters on the flexural and tensile strengths of neat ABS and ABS/CF composites using the larger-is-better criterion of the S/N ratio. For the flexural strength of neat ABS (Table 9 and Fig 12), the S/N ratio varies in the range of 15.66–15.95 dB. The delta (Δ) analysis indicates that melt temperature is the most influential factor (Δ = 0.27, Rank 1), followed by filling pressure (Δ = 0.25, Rank 2) and filling time (Δ = 0.23, Rank 3), whereas packing pressure exhibits the least influence (Δ = 0.12, Rank 5). These results demonstrate that, for neat ABS, the melt state of the polymer and the flow-filling behavior play dominant roles in determining the stability of flexural strength, as they strongly govern the development of microstructure and the formation of internal defects within the specimens.

**Table 12:** Response table for signal-to-noise ratios of tensile test for ABS/CF composite.

Level	Filling pressure(MPa)	Packing pressure(MPa)	Filling time (s)	Packing time (s)	Melt temperature(℃)
**1**	31.83	31.83	31.80	31.90	31.79
**2**	31.80	31.86	31.90	31.86	31.87
**3**	31.80	31.80	31.95	31.92	31.87
**4**	31.87	31.94	31.90	31.85	31.97
**5**	32.08	31.95	31.83	31.85	31.88
**Delta**	0.28	0.15	0.15	0.07	0.18
**Rank**	1	4	3	5	2

**Table 9:** Response table for signal-to-noise ratios of bending strength for ABS without CF.

Level	Filling pressure(MPa)	Packing pressure(MPa)	Filling time (s)	Packing time (s)	Melt temperature(℃)
**1**	15.70	15.75	15.66	15.67	15.70
**2**	15.80	15.77	15.86	15.84	15.78
**3**	15.70	15.83	15.89	15.77	15.67
**4**	15.72	15.82	15.78	15.82	15.80
**5**	15.95	15.71	15.69	15.79	15.93
**Delta**	0.25	0.12	0.23	0.17	0.27
**Rank**	2	5	3	4	1

In contrast, for the flexural strength of the ABS/CF composite (Table 10 and Fig 12), the S/N ratio increases markedly to a range of 15.86–16.59 dB, indicating higher mechanical stability compared with neat ABS. The most influential factor is filling pressure, with Δ = 0.73 (Rank 1), followed by packing pressure (Δ = 0.53, Rank 2) and melt temperature (Δ = 0.47, Rank 3). The two time-related parameters, namely filling time and packing time, exhibit lower contributions, with Δ values of 0.45 and 0.36, respectively. This shift in the ranking of influential factors suggests that, in the presence of the carbon fiber layer, pressure-related parameters become more critical because they directly govern the degree of melt impregnation into the fiber structure and the quality of the polymer–fiber interfacial bonding.

**Table 10:** Response table for signal-to-noise ratios of bending strength for ABS/CF composite.

Level	Filling pressure(MPa)	Packing pressure(MPa)	Filling time (s)	Packing time (s)	Melt temperature(℃)
**1**	15.86	15.90	16.26	16.24	16.24
**2**	16.13	16.34	16.28	16.38	16.03
**3**	16.24	16.23	15.95	16.10	16.23
**4**	16.48	16.43	16.40	16.46	16.29
**5**	16.59	16.40	16.41	16.12	16.50
**Delta**	0.73	0.53	0.45	0.36	0.47
**Rank**	1	2	4	5	3

A similar trend is also observed for tensile strength. For neat ABS (Table 11 and Fig 13), the S/N ratio ranges from 30.59 to 30.81 dB, with filling pressure remaining the most influential factor (Δ = 0.23, Rank 1), followed by packing pressure (Δ = 0.11, Rank 2) and packing time (Δ = 0.09, Rank 3). For the ABS/CF composite (Table 12 and Fig 13), the S/N ratio is noticeably higher (31.79–32.08 dB), and the ranking of influential parameters is filling pressure (Δ = 0.28, Rank 1)> melt temperature (Δ = 0.18, Rank 2)> filling time (Δ = 0.15, Rank 3), whereas packing time exhibits the least influence (Δ = 0.07, Rank 5). These results indicate that carbon fiber reinforcement not only increases the average tensile strength but also enhances the sensitivity of the mechanical properties to pressure- and temperature-related processing conditions during injection molding.

**Table 11:** Response table for signal-to-noise ratios of tensile test for ABS without CF.

Level	Filling pressure(MPa)	Packing pressure(MPa)	Filling time (s)	Packing time (s)	Melt temperature(℃)
**1**	30.59	30.66	30.62	30.64	30.64
**2**	30.58	30.69	30.67	30.69	30.67
**3**	30.64	30.59	30.69	30.66	30.64
**4**	30.69	30.66	30.64	30.70	30.71
**5**	30.81	30.70	30.69	30.61	30.65
**Delta**	0.23	0.11	0.07	0.09	0.08
**Rank**	1	2	5	3	4

A comparison between the two material systems reveals that the dominant mechanisms governing mechanical properties change markedly with the introduction of carbon fiber reinforcement: from a primary dependence on melt temperature in neat ABS to a strong dependence on pressure-related parameters in the ABS/CF composite. This finding is consistent with previous studies on fiber-reinforced thermoplastic composites, in which higher filling pressure enhances polymer impregnation into fiber bundles and improves stress transfer efficiency [5,32]. Furthermore, the higher and more stable S/N ratios observed for the ABS/CF composite are in agreement with the general conclusion that fiber-reinforced composites exhibit lower sensitivity to fluctuations in processing parameters compared with unreinforced polymers.

### 3.3. Mean value analysis

**Fig 14:**
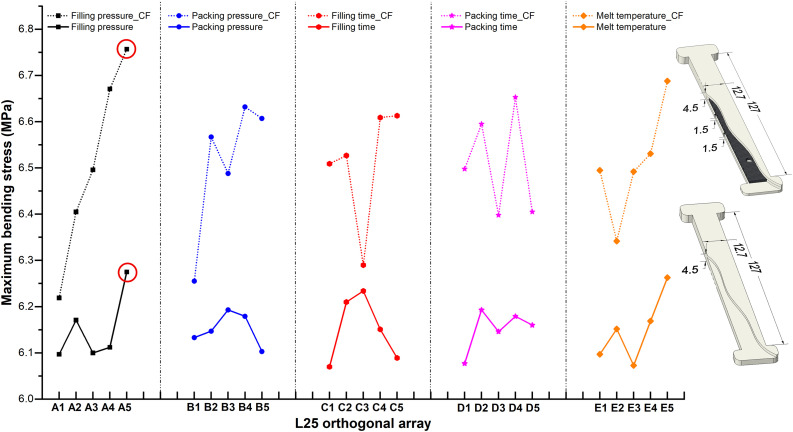
Main effects plots of mean bending strength for ABS and ABS/CF composite.

**Fig 15:**
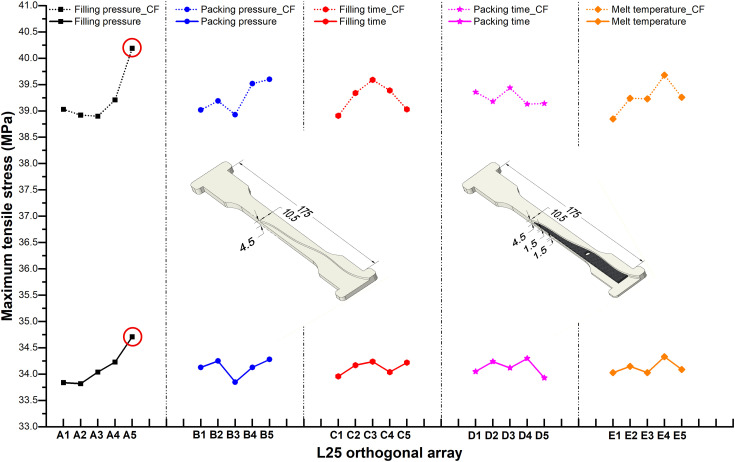
Mean tensile strength of ABS and ABS/CF composite under L25 orthogonal design.

Based on Tables 13 and 16 and the main effects plots (Figs 14 and 15), the influence of injection molding parameters on the mean flexural strength and mean tensile strength of neat ABS and ABS/CF composite was analyzed according to the larger-is-better criterion. For the mean flexural strength of neat ABS (Table 13), the obtained values ranged from 6.097 to 6.275 MPa. The range analysis (Δ) indicates that melt temperature is the most influential factor (Δ = 0.191, rank 1), followed by filling pressure (Δ = 0.179, rank 2) and filling time (Δ = 0.164, rank 3), whereas packing pressure exhibits the lowest influence (Δ = 0.090, rank 5). These results demonstrate that, for neat ABS, the thermal state of the polymer melt during injection molding plays a dominant role in the formation of a stable microstructure, which in turn directly governs the mean flexural strength of the final product.

**Table 13:** Response table for mean bending strength of ABS without CF.

Level	Filling pressure(MPa)	Packing pressure(MPa)	Filling time (s)	Packing time (s)	Melt temperature(℃)
**1**	6.097	6.133	6.070	6.077	6.097
**2**	6.171	6.147	6.210	6.193	6.152
**3**	6.100	6.193	6.234	6.146	6.073
**4**	6.112	6.179	6.151	6.179	6.169
**5**	6.275	6.103	6.089	6.160	6.263
**Delta**	0.179	0.090	0.164	0.115	0.191
**Rank**	2	5	3	4	1

**Table 16:** Response table for the mean tensile strength of ABS/CF composite.

Level	Filling pressure(MPa)	Packing pressure(MPa)	Filling time (s)	Packing time (s)	Melt temperature(℃)
**1**	39.03	39.02	38.91	39.36	38.85
**2**	38.92	39.19	39.34	39.18	39.24
**3**	38.90	38.93	39.59	39.44	39.23
**4**	39.21	39.52	39.39	39.13	39.68
**5**	40.19	39.60	39.03	39.14	39.26
**Delta**	1.30	0.67	0.68	0.31	0.83
**Rank**	1	4	3	5	2

In contrast, for the ABS/CF composite (Table 14), the mean flexural strength increases markedly, ranging from 6.219 to 6.757 MPa, which is approximately 5–8% higher than that of neat ABS. The most influential factor shifts to filling pressure (Δ = 0.537, rank 1), followed by packing pressure (Δ = 0.377, rank 2) and melt temperature (Δ = 0.346, rank 3). The two time-related parameters (filling time and packing time) exhibit only secondary effects (Δ = 0.323 and 0.255, respectively). This change in the ranking of factor influence indicates that, in the presence of the carbon fiber layer, pressure-related parameters become dominant because they govern the degree of resin impregnation into the fiber architecture and the quality of the fiber–matrix interfacial bonding.

**Table 14:** Response table of mean bending strength for ABS/CF composite.

Level	Filling pressure(MPa)	Packing pressure(MPa)	Filling time (s)	Packing time (s)	Melt temperature(℃)
**1**	6.219	6.255	6.509	6.498	6.495
**2**	6.405	6.567	6.527	6.595	6.342
**3**	6.496	6.488	6.290	6.398	6.492
**4**	6.671	6.632	6.609	6.653	6.531
**5**	6.757	6.607	6.613	6.405	6.688
**Delta**	0.537	0.377	0.323	0.255	0.346
**Rank**	1	2	4	5	3

For the mean tensile strength of neat ABS (Table 13), the average values range from 33.84 to 34.71 MPa. In this case, filling pressure remains the most dominant factor (Δ = 0.89, rank 1), followed by packing pressure (Δ = 0.43, rank 2), whereas filling time exhibits the weakest influence (Δ = 0.28, rank 5). When transitioning to the ABS/CF composite (Table 14), the mean tensile strength increases substantially to approximately 38.90–40.19 MPa, corresponding to an improvement of about 15–18% compared with neat ABS. The ranking of technological factors is as follows: filling pressure (Δ = 1.30, rank 1)> melt temperature (Δ = 0.83, rank 2)> filling time (Δ = 0.68, rank 3), while packing time shows the smallest effect (Δ = 0.31, rank 5).

A comparison between the two material systems indicates that carbon fiber reinforcement not only significantly increases the mean flexural and tensile strengths, but also alters the dominant processing mechanism, shifting from temperature-sensitive behavior in neat ABS to pressure-dominated behavior in the ABS/CF composite. This transition is consistent with previous studies, which reported that higher filling pressure enhances resin impregnation into the fiber layer and improves stress transfer across the fiber–matrix interface [32]. Moreover, the pronounced increase in mechanical properties of the ABS/CF composite can be attributed to the efficient stress transfer and load sharing between the high-modulus carbon fibers and the polymer matrix, resulting in improved stiffness and strength compared with neat polymers [5].

### 3.4. Taguchi–Grey Correlation analysis

After normalization of the S/N ratio data, the Grey relational coefficient (GRC) was calculated using Eq. (5), and the Grey relational grade (TGC) was subsequently obtained by averaging the GRC values for each experimental run. All parameter combinations were then ranked based on their TGC values, and the optimal processing condition was identified as the combination yielding the highest TGC, where values closer to 1 indicate superior multi-response performance (Tables 15,16).

**Table 15:** Response table for the mean tensile strength of ABS without CF.

Level	Filling pressure(MPa)	Packing pressure(MPa)	Filling time (s)	Packing time (s)	Melt temperature(℃)
**1**	33.84	34.13	33.96	34.05	34.03
**2**	33.82	34.25	34.17	34.24	34.15
**3**	34.04	33.85	34.24	34.12	34.03
**4**	34.23	34.13	34.04	34.30	34.33
**5**	34.71	34.28	34.22	33.93	34.09
**Delta**	0.89	0.43	0.28	0.36	0.30
**Rank**	1	2	5	3	4

**Fig 16:**
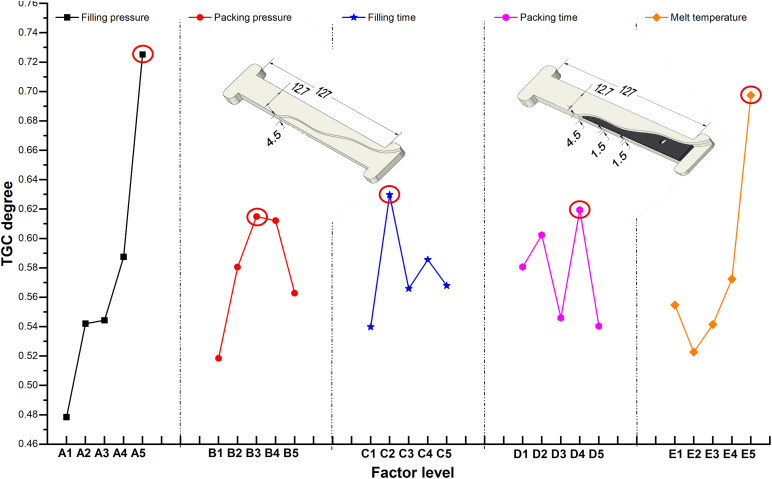
TGC degree of bending strength for each control factor at different levels.

**Fig 17:**
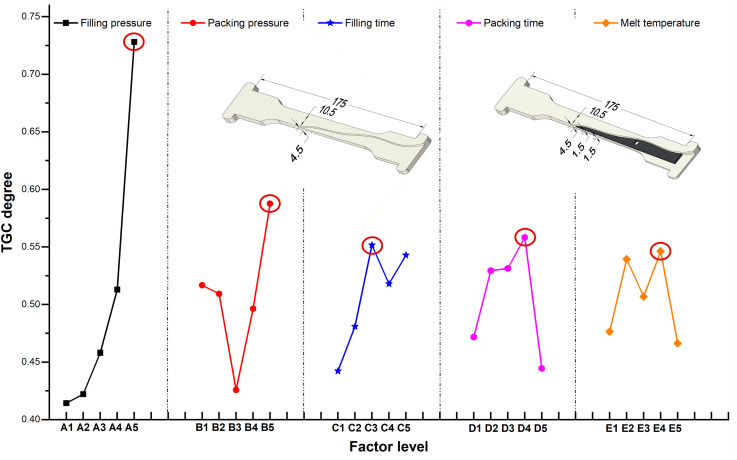
TGC degree of tensile strength for each control factor at different levels.

The calculated TGC values for flexural and tensile strengths are presented in Tables 17 and 18 and illustrated in Figs 16 and 17. The results clearly indicate that filling pressure consistently produces the highest TGC values across all processing parameters. Specifically, for flexural strength, the TGC value increases significantly from 0.478 at Level 1 to 0.725 at Level 5. A similar trend is observed for tensile strength, where the maximum TGC value reaches 0.728 at Level 5. These findings confirm that filling pressure is the primary governing parameter influencing the overall mechanical performance of the ABS/CF composite under multi-objective optimization conditions.

**Table 17:** TGC response table of bending strength.

Level	Filling pressure(MPa)	Packing pressure(MPa)	Filling time (s)	Packing time (s)	Melt temperature(℃)
**1**	0.478	0.518	0.539	0.580	0.554
**2**	0.542	0.580	0.629	0.602	0.522
**3**	0.555	0.614	0.565	0.546	0.541
**4**	0.587	0.612	0.585	0.619	0.572
**5**	0.725	0.562	0.567	0.540	0.697

**Table 18:** TGC response table of tensile strength.

Level	Filling pressure(MPa)	Packing pressure(MPa)	Filling time (s)	Packing time (s)	Melt temperature(℃)
**1**	0.414	0.516	0.442	0.471	0.476
**2**	0.422	0.509	0.480	0.529	0.539
**3**	0.458	0.425	0.551	0.531	0.506
**4**	0.512	0.496	0.517	0.558	0.546
**5**	0.728	0.587	0.542	0.444	0.466

The ranking of processing parameters based on TGC values further supports this observation. For flexural strength, the influence order can be summarized as: filling pressure > melt temperature > packing pressure > filling time > packing time. For tensile strength, a comparable trend is obtained, with filling pressure remaining dominant, followed by packing pressure, filling time, melt temperature, and packing time. This ranking is consistent with the ANOVA results, where filling pressure exhibits the lowest p-value and highest F-value among all factors, confirming its statistical significance and reinforcing the reliability of the combined Taguchi–Grey approach.

Melt temperature is identified as the second most influential factor for flexural strength, reaching a relatively high TGC value of 0.697 at Level 5. This suggests that elevated processing temperatures improve polymer chain mobility and melt flowability, thereby enhancing the impregnation of molten ABS into the carbon fiber layer and strengthening fiber–matrix interfacial bonding. In contrast, for tensile strength, packing pressure shows a more pronounced contribution, achieving a maximum TGC value of 0.587 at Level 5, indicating its role in improving material densification and stress transfer efficiency.

The dominant role of filling pressure can be attributed to its strong influence on melt flow behavior and fiber impregnation during the insert injection molding process. Higher filling pressure promotes deeper penetration of the molten polymer into the carbon fiber network, resulting in improved wetting, reduced void content, and enhanced interfacial bonding between the matrix and reinforcement. These findings are in good agreement with previous studies, which reported that increasing injection pressure significantly improves mechanical properties through enhanced fiber–matrix adhesion and reduced internal defects [34].

Similarly, the observed influence of melt temperature on flexural strength aligns with literature reports indicating that appropriate thermal conditions enhance resin flowability and fiber wetting, thereby improving composite performance [5]. However, excessively high temperatures may lead to matrix degradation or interfacial weakening, which explains the moderate variation of TGC values across temperature levels observed in this study.

Compared with neat ABS, the ABS/CF composite exhibits a stronger sensitivity to pressure-related parameters, indicating a transition in the governing mechanism from thermally dominated behavior in pure polymers to flow- and compaction-controlled behavior in fiber-reinforced systems. This observation is consistent with previous studies on layered and hybrid composites processed via injection molding, where pressure plays a decisive role in controlling fiber distribution, impregnation quality, and ultimately the mechanical performance of the final product [32].

To validate the optimal parameter combination obtained from the Taguchi–Grey analysis, a confirmation experiment was conducted using the predicted optimal processing parameters. The predicted and experimental results are summarized in Table 19. The experimental results show good agreement with the predicted values, with deviations of 1.59% for flexural strength and 2.98% for tensile strength, respectively, both of which are below 5%. This confirms the reliability and effectiveness of the proposed optimization approach.

**Table 19:** Confirmation experiment results for optimal processing parameters.

Response	Predicted value (MPa)	Experimental value (MPa)	Deviation (%)
Flexural strength (ABS/CF)	6.93	6.82	1.59
Tensile strength (ABS/CF)	41.27	40.04	2.98

Overall, the GRA results not only confirm the effectiveness of the Taguchi–Grey method for simultaneous multi-objective optimization but also provide a clear physical interpretation of the processing–structure–property relationship in ABS/CF composites. The consistency between GRA and ANOVA analyses highlights that pressure-related parameters, particularly filling pressure, should be prioritized, in combination with an appropriate melt temperature, to achieve optimal mechanical performance in injection-molded fiber-reinforced thermoplastic composites.

## 4. Conclusion

This study systematically evaluated the effects of key injection molding parameters, including filling pressure, packing pressure, filling time, packing time, and melt temperature, on the flexural and tensile properties of neat ABS and ABS/CF composites using a Taguchi L25 design combined with S/N analysis and Grey relational analysis (GRA).

The results show that the incorporation of carbon fiber significantly enhances the mechanical performance of ABS. The maximum flexural strength increased from 6.49 MPa for neat ABS to 6.93 MPa for ABS/CF composites, while the maximum tensile strength increased from 35.17 MPa to 41.27 MPa, corresponding to an improvement of approximately 17–18%.

Statistical analysis indicates that filling pressure is the most dominant factor affecting both flexural and tensile properties. For ABS/CF composites, filling pressure and packing pressure exhibit statistically significant effects under flexural loading, with p-values of 0.012 and 0.039 (p < 0.05), respectively. In terms of mean response analysis, filling pressure shows the highest Delta values (0.73 for flexural strength and 1.30 for tensile strength), confirming its governing role. The overall influence ranking for ABS/CF composites is determined as: filling pressure (A)> packing pressure (B)> melt temperature (E)> filling time (C)> packing time (D).

The Taguchi–Grey analysis successfully identifies the optimal parameter combination, yielding maximum Grey relational grades of 0.725 for flexural strength and 0.728 for tensile strength. Confirmation experiments demonstrate good agreement between predicted and experimental results, with deviations of less than 3%, confirming the reliability of the optimization approach.

Overall, the results demonstrate that pressure-related parameters play a critical role in controlling melt flow behavior, fiber impregnation, and interfacial bonding, thereby governing the mechanical performance of ABS/CF composites. The combined Taguchi–Grey approach provides an effective method for multi-objective optimization and offers practical guidance for improving the performance of injection-molded fiber-reinforced thermoplastic components.
