# Supplementary material for: Optimization of injection molding parameters for bending and tensile strength of ABS and ABS/carbon fiber composites using Taguchi–Grey correlation analysis
Source: PLoS One. 2026 Jul 15;21(7):e0352647. doi: 10.1371/journal.pone.0352647 (PMC13372186; doi:10.1371/journal.pone.0352647)
Supplement: Attachment — Submitted filename: Plos one_Responses to reviewer #4.docx [file pone.0352647.s002.docx]

Dear **Reviewer #4**,

Thank you very much for your continued review and constructive comments. We carefully revised the manuscript according to your suggestions. Below we provide point-by-point responses, together with the exact locations of the revisions in the new version.

**Comment 1:** *Add recent literature.*

**Response:** Thank you for this valuable suggestion. The manuscript has been revised to incorporate recent literature relevant to injection-molded fiber-reinforced thermoplastic composites and process optimization methods.

Specifically, several recent studies (published within the last 3–5 years) have been added to the Introduction and Discussion sections to highlight key findings related to processing parameters, fiber–matrix interaction, and mechanical performance. These additions help strengthen the context of the study and better position the research within the current state of the art.

**Comment 2:** *Compare the results with the published papers.*

**Response:** Thank you for this valuable comment. The manuscript has been revised to include a detailed comparison of the obtained results with previously published studies.

Specifically, the flexural and tensile strengths, percentage improvements, and the influence of processing parameters have been compared with relevant literature on injection-molded fiber-reinforced thermoplastic composites. These comparisons have been incorporated in the Results and Discussion section to better highlight the consistency and differences between the present study and existing works.

The revised content can be found in the manuscript (**pp. 19–33**).

**Comment 3:** *Delete the unnecessary references.*

**Response:** Thank you for this valuable comment. The reference list has been carefully reviewed and revised to remove unnecessary or redundant citations.

Specifically, overly generic references, outdated sources, and those not directly relevant to the scope of injection-molded fiber-reinforced thermoplastic composites have been removed. The remaining references have been refined to emphasize recent and highly relevant studies, particularly those reporting quantitative findings and related methodologies.

The updated reference list is presented in the revised manuscript (**pp. 35–38**).

**Comment 4:** *Inclue the schematic work flow diagram.*

**Response:** Thank you for this valuable suggestion. A schematic workflow diagram has been added to the revised manuscript to clearly illustrate the overall research methodology.

The diagram summarizes the key steps of the study, including experimental design (Taguchi L25), injection molding process, mechanical testing (tensile and flexural), and subsequent statistical analyses (S/N ratio, Grey relational analysis, and ANOVA), as well as the optimization and confirmation stages.

The added figure can be found in the revised manuscript (**Figure 1, p. 4**).

**Comment 5:** *Conclusions should be modified with key results.*

**Response:** Thank you for this valuable suggestion. The conclusion section has been revised to include key quantitative and technical results to strengthen its clarity and impact.

Specifically, numerical values related to mechanical performance (maximum flexural and tensile strengths), percentage improvements, statistical significance (p-values), contribution analysis, and optimization results (Grey relational grades and confirmation experiment deviations) have been incorporated into the revised conclusion (**pp. 34–35**).

We appreciate your insightful comments, which have substantially improved the manuscript’s clarity and rigor. We attach the revised version (clean and tracked) together with this response letter.

***With best regards,***
on behalf of all co-authors
Faculty of Mechanical Engineering, HCM-UTE
