# Supplementary material for: Optimization of injection molding parameters for bending and tensile strength of ABS and ABS/carbon fiber composites using Taguchi–Grey correlation analysis
Source: PLoS One. 2026 Jul 15;21(7):e0352647. doi: 10.1371/journal.pone.0352647 (PMC13372186; doi:10.1371/journal.pone.0352647)
Supplement: Peer review history

# Peer review: Decision Letter 0

**DOI:** 10.1371/journal.pone.0352647.r001

**Authors:** Vijay Raghunathan

13 Mar 2026

PONE-D-26-07993

Optimization of injection molding parameters for bending and tensile strength of ABS and ABS/carbon fiber hybrid composites using Taguchi–Grey correlation analysis

PLOS One

Dear Dr. Son Minh,

Thank you for submitting your manuscript to PLOS ONE. After careful consideration, we feel that it has merit but does not fully meet PLOS ONE’s publication criteria as it currently stands. Therefore, we invite you to submit a revised version of the manuscript that addresses the points raised during the review process.

Please submit your revised manuscript by Apr 27 2026 11:59PM. If you will need more time than this to complete your revisions, please reply to this message or contact the journal office at plosone@plos.org. When you're ready to submit your revision, log on to https://www.editorialmanager.com/pone/ and select the 'Submissions Needing Revision' folder to locate your manuscript file.

Please include the following items when submitting your revised manuscript:

- A letter that responds to each point raised by the academic editor and reviewer(s). You should upload this letter as a separate file labeled 'Response to Reviewers'.
- A marked-up copy of your manuscript that highlights changes made to the original version. You should upload this as a separate file labeled 'Revised Manuscript with Track Changes'.
- An unmarked version of your revised paper without tracked changes. You should upload this as a separate file labeled 'Manuscript'.

If you would like to make changes to your financial disclosure, please include your updated statement in your cover letter. Guidelines for resubmitting your figure files are available below the reviewer comments at the end of this letter.

If applicable, we recommend that you deposit your laboratory protocols in protocols.io to enhance the reproducibility of your results. Protocols.io assigns your protocol its own identifier (DOI) so that it can be cited independently in the future. For instructions see: https://journals.plos.org/plosone/s/submission-guidelines#loc-laboratory-protocols. Additionally, PLOS ONE offers an option for publishing peer-reviewed Lab Protocol articles, which describe protocols hosted on protocols.io. Read more information on sharing protocols at https://plos.org/protocols?utm_medium=editorial-email&utm_source=authorletters&utm_campaign=protocols.

We look forward to receiving your revised manuscript.

Kind regards,

Vijay Raghunathan

Academic Editor

PLOS One

Journal Requirements:

When submitting your revision, we need you to address these additional requirements.

1. Please ensure that your manuscript meets PLOS ONE's style requirements, including those for file naming. The PLOS ONE style templates can be found at

https://journals.plos.org/plosone/s/file?id=wjVg/PLOSOne_formatting_sample_main_body.pdf and https://journals.plos.org/plosone/s/file?id=ba62/PLOSOne_formatting_sample_title_authors_affiliations.pdf

2. Please update your submission to use the PLOS LaTeX template. The template and more information on our requirements for LaTeX submissions can be found at http://journals.plos.org/plosone/s/latex.

3. We note that Figure(s) 2 - 17 in your submission contain copyrighted images. All PLOS content is published under the Creative Commons Attribution License (CC BY 4.0), which means that the manuscript, images, and Supporting Information files will be freely available online, and any third party is permitted to access, download, copy, distribute, and use these materials in any way, even commercially, with proper attribution. For more information, see our copyright guidelines: http://journals.plos.org/plosone/s/licenses-and-copyright.

We require you to either (1) present written permission from the copyright holder to publish these figures specifically under the CC BY 4.0 license, or (2) remove the figures from your submission:

a. You may seek permission from the original copyright holder of Figure(s) 2 - 17 to publish the content specifically under the CC BY 4.0 license.

We recommend that you contact the original copyright holder with the Content Permission Form (http://journals.plos.org/plosone/s/file?id=7c09/content-permission-form.pdf) and the following text:

“I request permission for the open-access journal PLOS ONE to publish XXX under the Creative Commons Attribution License (CCAL) CC BY 4.0 (http://creativecommons.org/licenses/by/4.0/). Please be aware that this license allows unrestricted use and distribution, even commercially, by third parties. Please reply and provide explicit written permission to publish XXX under a CC BY license and complete the attached form.”

Please upload the completed Content Permission Form or other proof of granted permissions as an "Other" file with your submission.

In the figure caption of the copyrighted figure, please include the following text: “Reprinted from [ref] under a CC BY license, with permission from [name of publisher], original copyright [original copyright year].”

b. If you are unable to obtain permission from the original copyright holder to publish these figures under the CC BY 4.0 license or if the copyright holder’s requirements are incompatible with the CC BY 4.0 license, please either i) remove the figure or ii) supply a replacement figure that complies with the CC BY 4.0 license. Please check copyright information on all replacement figures and update the figure caption with source information. If applicable, please specify in the figure caption text when a figure is similar but not identical to the original image and is therefore for illustrative purposes only.

4. If the reviewer comments include a recommendation to cite specific previously published works, please review and evaluate these publications to determine whether they are relevant and should be cited. There is no requirement to cite these works unless the editor has indicated otherwise.

Additional Editor Comments :

Based on the reviewer's recommendations, Manuscript is recommended for Major revision. It could be seen that some reviewer's have asked to cite certain papers. It is authors wish to cite any papers asked by reviewer's only if it is relevant to current work. The final decision is not influenced by citing.

[Note: HTML markup is below. Please do not edit.]

Reviewers' comments:

Reviewer's Responses to Questions

**Comments to the Author**

1. Is the manuscript technically sound, and do the data support the conclusions?

The manuscript must describe a technically sound piece of scientific research with data that supports the conclusions. Experiments must have been conducted rigorously, with appropriate controls, replication, and sample sizes. The conclusions must be drawn appropriately based on the data presented.

Reviewer #1: Yes

Reviewer #2: Yes

Reviewer #3: No

Reviewer #4: Yes

**********

2. Has the statistical analysis been performed appropriately and rigorously?

Reviewer #1: Yes

Reviewer #2: Yes

Reviewer #3: Yes

Reviewer #4: Yes

**********

3. Have the authors made all data underlying the findings in their manuscript fully available?

The PLOS Data policy requires authors to make all data underlying the findings described in their manuscript fully available without restriction, with rare exception (please refer to the Data Availability Statement in the manuscript PDF file). The data should be provided as part of the manuscript or its supporting information, or deposited to a public repository. For example, in addition to summary statistics, the data points behind means, medians and variance measures should be available. If there are restrictions on publicly sharing data—e.g. participant privacy or use of data from a third party—those must be specified.

Reviewer #1: Yes

Reviewer #2: Yes

Reviewer #3: Yes

Reviewer #4: Yes

**********

4. Is the manuscript presented in an intelligible fashion and written in standard English?

PLOS ONE does not copyedit accepted manuscripts, so the language in submitted articles must be clear, correct, and unambiguous. Any typographical or grammatical errors should be corrected at revision, so please note any specific errors here.

Reviewer #1: Yes

Reviewer #2: Yes

Reviewer #3: No

Reviewer #4: Yes

**********

5. Review Comments to the Author

Please use the space provided to explain your answers to the questions above. You may also include additional comments for the author, including concerns about dual publication, research ethics, or publication ethics. (Please upload your review as an attachment if it exceeds 20,000 characters)

Reviewer #1: 1.Authors need to add more quantitative results in the abstract section.

2.The reported maximum flexural strength for ABS is 6.49 MPa and for ABS/CF is 6.93 MPa. This is extremely low compared to typical ABS flexural strength values (generally 60–90 MPa). The values appear to be off by approximately one order of magnitude. This must be carefully rechecked, as it directly affects the validity of the results.

3.The tensile strength of neat ABS ranges 33–35 MPa, while ABS/CF reaches 38–41 MPa. An 18% improvement is reported. For a woven carbon fabric reinforcement layer (0.36 mm thickness), the improvement is relatively modest. The authors should provide fiber volume fraction calculation and explain why improvement is not higher despite continuous fiber reinforcement.

4.Page 11 states that placing the carbon fiber layer at the neutral plane enhances load-bearing performance. In bending, the neutral axis experiences zero normal stress. Reinforcement is most effective when placed away from the neutral axis.

5.In Table 4 (p. 20), experiment No. 4 lists packing pressure = 33 MPa. All other packing pressures are between 96–100 MPa. This is likely a typographical error. This must be corrected and recalculated if necessary.

6.Units formatting inconsistent (mm/phút). Vietnamese word “phút” appears in Eq. (6)

7.Calculated bending speed = 1.92 mm/min, but actual testing performed at 15 mm/min due to machine limitation. This is nearly 8× higher than ASTM-recommended rate. The authors must justify this deviation and discuss its effect.

8.Grey Relational Analysis: A confirmation experiment is necessary to validate optimization.

9.Add suitable references for the mentioned ASTM standards. The Introduction would benefit from adding a brief statement highlighting the successful application of the optimization methods (Taguchi, RSM, etc.) across diverse engineering fields, supported by following citations; Authors can cite following article in Introduction, methodology and Discussion sections;

ASTM D790: Effect of reinforced weave patterns on the mechanical performance and wear resistance of wool-epoxy composites.

Experimental and Computational Analysis of Epoxy-Cotton Seed Particle Composites: A Multi-Property Study for Automotive Use.

ASTM D638: Machine learning based prediction on mechanical and wear characterization of sisal fiber reinforced dolomite dust-epoxy hybrid composites

Tribological study on slurry abrasive wear behavior of nonwoven viscose fabric composites with doe approach.

Multi-objective optimization and experimental analysis of electro-discharge machining parameters via Gray-Taguchi, TOPSIS-Taguchi and PSI-Taguchi methods.

Analysis of sliding wear behavior of LD sludge filled epoxy composites using response surface methodology.

Analysis of erosion wear behavior of LD sludge filled polypropylene composites using RSM.

Computational modelling and optimization of mechanical properties of red brick dust‐epoxy composites using machine learning approach.

Machine learning guided optimization of engineered wood dust reinforced hybrid polymer composites.

10.Table 1: ABS is thermoplastic; it does not cure. Replace with “processing temperature” or remove.

11.Table 2: “Single-fiber tensile strength = 4000 MPa” needs citation.

12.Tables 5 & 6: No standard deviation reported. Since 3 replicates were tested, mean ± SD should be provided.

13.ANOVA: R2, F-values and p-values not clearly reported.

14.0.01mm/mm/min ............. give a space between value and unit, and do similar corrections throughout the manuscript.

15.Add technical facts to the conclusions to improve them.

Reviewer #2: The research article is interesting the statistical part is good. Include the most recent papers in the reference section and conclusion needs to be written to the point of the findings. All the very best.

Reviewer #3: I have reviewed the article titled "Optimization of injection molding parameters for bending and tensile strength of ABS and ABS/carbon fiber hybrid composites using Taguchi–Grey correlation analysis". The article does not present any new findings other than the use of statistical analysis; therefore, I don't recommend for publication. The authors may consider comments below for improvement

1) In the Title is ABS-CF hybrid composite is misleading, only if one or more reinforcements were used, it can be termed as hybrid composite

2) Introduction is generic, authors have to discuss mainly about the findings from the recently published works of similar scope, identify research gaps and indicate the importance of the study. Remove references cited on generic statements and include findings

3) How was the parameter range selected for injection moulding?

4) Equation 6 unit is not SI

5) Section 2.4 and 2.7 must be merged

6) For tensile and flexural tests, stress - strain data must be included. How many specimens tested in each category?

7) SEM Microstructure of fractured specimens from tensile test should be included

8) Grey relational analysis is incomplete. Further from S/N ratio, grey relational coefficient (GRC) and ranking must be obtained. GRC close to 1 is optimum combination

Reviewer #4: 1. Add recent literature.

2. Compare the results with the published papers

3. Delete the unnecessary references.

4. Inclue the schematic work flow diagram..

5. Conclusions should be modified with key results.

**********

6. PLOS authors have the option to publish the peer review history of their article (what does this mean?). If published, this will include your full peer review and any attached files.

If you choose “no”, your identity will remain anonymous but your review may still be made public.

**Do you want your identity to be public for this peer review?**  For information about this choice, including consent withdrawal, please see our Privacy Policy.

Reviewer #1: No

Reviewer #2: No

Reviewer #3: **Yes:** Chandrasekar Muthukumar

Reviewer #4: **Yes:** NARAYANASAMY PANDIARAJAN

**********

[NOTE: If reviewer comments were submitted as an attachment file, they will be attached to this email and accessible via the submission site. Please log into your account, locate the manuscript record, and check for the action link "View Attachments". If this link does not appear, there are no attachment files.]

To ensure your figures meet our technical requirements, please review our figure guidelines: https://journals.plos.org/plosone/s/figures

You may also use PLOS’s free figure tool, NAAS, to help you prepare publication quality figures: https://journals.plos.org/plosone/s/figures#loc-tools-for-figure-preparation.

NAAS will assess whether your figures meet our technical requirements by comparing each figure against our figure specifications.

# Author comment: Author response to Decision Letter 1

**DOI:** 10.1371/journal.pone.0352647.r002

15 May 2026

Dear Reviewer #1,

Thank you very much for your continued review and constructive comments. We carefully revised the manuscript according to your suggestions. Below we provide point-by-point responses, together with the exact locations of the revisions in the new version.

Comment 1: Authors need to add more quantitative results in the abstract section.

Response: Thank you for this valuable suggestion. The abstract has been revised to include key quantitative results to improve its clarity and impact.

Specifically, numerical values such as the maximum flexural strength (6.49 MPa for ABS and 6.93 MPa for ABS/CF), maximum tensile strength (35.17 MPa for ABS and 41.27 MPa for ABS/CF), percentage improvement (~17–20%), and key optimization results have been incorporated into the revised abstract (p. 1).

Comment 2: The reported maximum flexural strength for ABS is 6.49 MPa and for ABS/CF is 6.93 MPa. This is extremely low compared to typical ABS flexural strength values (generally 60–90 MPa). The values appear to be off by approximately one order of magnitude. This must be carefully rechecked, as it directly affects the validity of the results.

Response: Thank you for this important comment. We have carefully rechecked the reported flexural strength values and confirm that the results are correct as presented.

The relatively low flexural strength values are attributed to the specific specimen configuration and testing conditions used in this study. In particular, the hybrid ABS/CF structure contains a thin carbon fiber fabric layer (≈ 0.36 mm) embedded at the mid-plane, while the overall specimen thickness is 4.5 mm. Under bending, the outer polymer layers (ABS) primarily experience the maximum tensile and compressive stresses, whereas the reinforcement located near the mid-plane contributes less effectively to flexural strength.

In addition, the interfacial bonding between the ABS matrix and the carbon fiber fabric, as well as the layered structure, may introduce stress concentration and limit load transfer efficiency under bending conditions. These factors collectively result in lower measured flexural strength compared to bulk ABS values reported in the literature.

To avoid confusion, we have clarified this explanation in the revised manuscript and emphasized the influence of specimen geometry and reinforcement configuration on the bending performance.

Comment 3: The tensile strength of neat ABS ranges 33–35 MPa, while ABS/CF reaches 38–41 MPa. An 18% improvement is reported. For a woven carbon fabric reinforcement layer (0.36 mm thickness), the improvement is relatively modest. The authors should provide fiber volume fraction calculation and explain why improvement is not higher despite continuous fiber reinforcement.

Response: We thank the reviewer for this valuable comment. Although a continuous woven carbon fiber layer was employed, the observed improvement in tensile strength (~15–18%) is indeed moderate. This can be explained by the relatively low fiber volume fraction and the specific structural configuration of the composite.

In addition, the injection molding process introduces several factors that may reduce the effectiveness of the reinforcement, including:

• incomplete resin impregnation into the woven fiber architecture,

• interfacial imperfections between fiber and matrix,

• and potential fiber waviness or local misalignment.

These factors collectively reduce stress transfer efficiency between the fiber and matrix.

Therefore, the observed improvement (~15–18%) is considered reasonable for a hybrid structure with a relatively low fiber volume fraction and a mid-plane reinforcement configuration.

Comment 4: Page 11 states that placing the carbon fiber layer at the neutral plane enhances load-bearing performance. In bending, the neutral axis experiences zero normal stress. Reinforcement is most effective when placed away from the neutral axis.

Response: Thank you for this insightful comment. We fully agree that, in bending, the neutral axis experiences zero normal stress, and reinforcement is generally more effective when positioned away from the neutral axis to maximize load-bearing capacity.

In the revised manuscript, all related statements have been corrected to remove the inaccurate implication that placing the carbon fiber layer at the neutral plane enhances load-bearing performance. Instead, the role of the carbon fiber layer positioned near the mid-plane is clarified as improving structural symmetry, interfacial bonding, and resistance to interlaminar delamination, rather than directly contributing to bending stress resistance.

The discussion has been revised throughout the manuscript to ensure consistency with the correct mechanical interpretation of stress distribution in bending.

Comment 5: In Table 4 (p. 20), experiment No. 4 lists packing pressure = 33 MPa. All other packing pressures are between 96–100 MPa. This is likely a typographical error. This must be corrected and recalculated if necessary.

Response: Thank you for your careful observation. The packing pressure value reported for experiment No. 4 was indeed a typographical error. The incorrect value “33 MPa” has been corrected to “99 MPa” in the revised manuscript (Table 4, p. 16).

We have thoroughly verified the dataset and confirm that this correction does not affect the Taguchi design matrix, S/N ratio calculations, or the overall statistical analysis and conclusions. All related calculations were rechecked to ensure consistency and accuracy.

The revised Table 4 has been updated accordingly in the manuscript (p. 16).

Comment 6: Units formatting inconsistent (mm/phút). Vietnamese word “phút” appears in Eq. (6)

Response: Thank you for your careful observation. The unit formatting has been corrected throughout the manuscript. Specifically, the Vietnamese term “mm/phút” in Eq. (6) has been revised to the standard SI unit format “mm/min” (p. 17).

We have also carefully reviewed the entire manuscript to ensure consistency in unit formatting across all equations, tables, and figures.

Comment 7: Calculated bending speed = 1.92 mm/min, but actual testing performed at 15 mm/min due to machine limitation. This is nearly 8× higher than ASTM-recommended rate. The authors must justify this deviation and discuss its effect.

Response: Thank you for this valuable comment.

The theoretically calculated displacement rate for the flexural test was 1.92 mm/min according to ASTM D790. However, due to limitations of the available testing machine, the experiments were conducted at a constant crosshead speed of 15 mm/min.

It is acknowledged that this value is higher than the ASTM-recommended rate. Nevertheless, this deviation was consistently applied to all tested specimens, including both neat ABS and ABS/CF composites, ensuring that the comparative analysis between different material systems and processing conditions remains valid.

In addition, previous studies have reported that, within a moderate range of loading rates, the flexural strength of thermoplastic composites is not significantly affected, although slight variations in ductility and strain at break may occur. The primary trends and relative performance differences between samples are therefore preserved under a constant testing condition.

Moreover, the main objective of this study is to evaluate the influence of injection molding parameters and to perform multi-objective optimization using Taguchi–Grey analysis. Since all experiments were conducted under identical testing conditions, the reliability of the optimization results and parameter ranking is not compromised.

A clarification regarding the testing speed and its potential influence has been added to the revised manuscript.

Comment 8: Grey Relational Analysis: A confirmation experiment is necessary to validate optimization.

Response: Thank you for this important comment. A confirmation experiment has been conducted to validate the optimal parameter combination obtained from the Taguchi–Grey relational analysis.

The results of the confirmation experiment have been added in the revised manuscript (pp. 31–32, Section 3.4). The experimental results show good agreement with the predicted values, with deviations of 1.59% for flexural strength and 2.98% for tensile strength, confirming the reliability and effectiveness of the proposed optimization approach.

Comment 9: Add suitable references for the mentioned ASTM standards. The Introduction would benefit from adding a brief statement highlighting the successful application of the optimization methods (Taguchi, RSM, etc.) across diverse engineering fields, supported by following citations; Authors can cite following article in Introduction, methodology and Discussion sections;

ASTM D790: Effect of reinforced weave patterns on the mechanical performance and wear resistance of wool-epoxy composites.

Experimental and Computational Analysis of Epoxy-Cotton Seed Particle Composites: A Multi-Property Study for Automotive Use.

ASTM D638: Machine learning based prediction on mechanical and wear characterization of sisal fiber reinforced dolomite dust-epoxy hybrid composites

Tribological study on slurry abrasive wear behavior of nonwoven viscose fabric composites with doe approach.

Multi-objective optimization and experimental analysis of electro-discharge machining parameters via Gray-Taguchi, TOPSIS-Taguchi and PSI-Taguchi methods.

Analysis of sliding wear behavior of LD sludge filled epoxy composites using response surface methodology.

Analysis of erosion wear behavior of LD sludge filled polypropylene composites using RSM.

Computational modelling and optimization of mechanical properties of red brick dust‐epoxy composites using machine learning approach.

Machine learning guided optimization of engineered wood dust reinforced hybrid polymer composites.

Response: Thank you for your valuable suggestion. Suitable references for the mentioned ASTM standards have been added, and additional recent studies demonstrating the successful application of optimization methods (Taguchi, RSM, and hybrid approaches) across various engineering fields have been incorporated.

Specifically, a brief statement highlighting the effectiveness of these optimization methods has been included in the Introduction (pp. 2–3), supported by the recommended and related references. These references have also been appropriately cited in the methodology and discussion sections where relevant.

Furthermore, the reference list has been updated accordingly (pp. 35–38) to ensure completeness and consistency with the revised manuscript.

Comment 10: Table 1: ABS is thermoplastic; it does not cure. Replace with “processing temperature” or remove.

Response: Thank you for your valuable comment. We acknowledge that ABS is a thermoplastic polymer and does not involve a curing reaction. Therefore, the term “curing temperature” was inappropriate.

In the revised manuscript, this term has been removed and replaced with “melt temperature” to accurately reflect the processing conditions (Table 1, p. 6). The table has been updated accordingly to ensure consistency with the material behavior.

Comment 11: Table 2: “Single-fiber tensile strength = 4000 MPa” needs citation.

Response: Thank you for this valuable comment. The value of single-fiber tensile strength (4000 MPa) has now been properly referenced in the revised manuscript (Table 2, p. 6).

Specifically, this value is supported by both the manufacturer’s technical datasheet and relevant literature. For example, the tensile strength of TR30 carbon fibers is reported to be approximately 4.12 GPa (≈ 4000 MPa) [28], which is consistent with the value used in this study.

Comment 12: Tables 5 & 6: No standard deviation reported. Since 3 replicates were tested, mean ± SD should be provided.

Response: Thank you for this valuable comment. The results in Tables 5 and 6 have been revised to include standard deviation values.

All mechanical properties are now presented in the format of mean ± standard deviation (n = 3), as recommended. The updated tables have been incorporated in the revised manuscript (pp. 20–22).

Comment 13: ANOVA: R2, F-values and p-values not clearly reported.

Response: Thank you for this important comment. The ANOVA results have been revised to clearly report the statistical parameters, including R² values, F-values, and p-values.

These indicators are now explicitly presented in the revised manuscript (pp. 23–24), both in the ANOVA tables and in the corresponding discussion to improve clarity and interpretation of the statistical significance and model adequacy.

Comment 14: 0.01 mm/mm/min ............. give a space between value and unit, and do similar corrections throughout the manuscript.

Response: Thank you for your careful observation. The spacing between numerical values and units has been corrected in the revised manuscript. Specifically, expressions such as “0.01mm/mm/min” have been revised to “0.01 mm/mm/min” (p. 17).

In addition, the entire manuscript has been carefully reviewed to ensure consistent formatting of values and units throughout all equations, tables, and text.

Comment 15: Add technical facts to the conclusions to improve them.

Response: Thank you for this valuable suggestion. The conclusion section has been revised to include key quantitative and technical results to strengthen its clarity and impact.

Specifically, numerical values related to mechanical performance (maximum flexural and tensile strengths), percentage improvements, statistical significance (p-values), contribution analysis, and optimization results (Grey relational grades and confirmation experiment deviations) have been incorporated into the revised conclusion (pp. 34–35).

We appreciate your insightful comments, which have substantially improved the manuscript’s clarity and rigor. We attach the revised version (clean and tracked) together with this response letter.

With best regards,

On behalf of all co-authors

Faculty of Mechanical Engineering, HCM-UTE

-------------------------------------------------------------------------------------------------------------------------------------------------

Dear Reviewer #2,

Thank you for your positive and constructive feedback.

Recent references have been added to the manuscript to strengthen the literature background. In addition, the conclusion section has been revised to more clearly and concisely present the key findings of the study.

With best regards,

On behalf of all co-authors

Faculty of Mechanical Engineering, HCM-UTE

-------------------------------------------------------------------------------------------------------------------------------------------------

Dear Reviewer #3,

Thank you very much for your continued review and constructive comments. We carefully revised the manuscript according to your suggestions. Below we provide point-by-point responses, together with the exact locations of the revisions in the new version.

Comment 1: In the Title is ABS-CF hybrid composite is misleading, only if one or more reinforcements were used, it can be termed as hybrid composite.

Response: Thank you for this valuable comment. We agree that the term “hybrid composite” may be misleading in this context, as only a single type of reinforcement (carbon fiber) was used.

Accordingly, the title has been revised to remove the term “hybrid” and now accurately reflects the material system studied (p. 1).

Comment 2: Introduction is generic, authors have to discuss mainly about the findings from the recently published works of similar scope, identify research gaps and indicate the importance of the study. Remove references cited on generic statements and include findings

Response: Thank you for this insightful comment. The Introduction has been thoroughly revised to address the reviewer’s concerns.

Specifically, recent studies related to injection-molded fiber-reinforced composites and optimization methods (e.g., Taguchi, RSM, and hybrid approaches) have been incorporated with a focus on their key findings rather than generic descriptions. In addition, a clear research gap has been identified regarding the limited understanding of the process–structure–property relationship in ABS/carbon fiber systems under injection moldi

Attachment Submitted filename: Plos one_Responses to reviewer #4.docx

# Peer review: Decision Letter 1

**DOI:** 10.1371/journal.pone.0352647.r003

**Authors:** Vijay Raghunathan

14 Jun 2026

Optimization of injection molding parameters for bending and tensile strength of ABS and ABS/carbon fiber composites using Taguchi–Grey correlation analysis

PONE-D-26-07993R1

Dear Dr. Son Minh,

We’re pleased to inform you that your manuscript has been judged scientifically suitable for publication and will be formally accepted for publication once it meets all outstanding technical requirements.

Within one week, you’ll receive an e-mail detailing the required amendments. When these have been addressed, you’ll receive a formal acceptance letter and your manuscript will be scheduled for publication.

An invoice will be generated when your article is formally accepted. Please note, if your institution has a publishing partnership with PLOS and your article meets the relevant criteria, all or part of your publication costs will be covered. Please make sure your user information is up-to-date by logging into Editorial Manager at Editorial Manager® and clicking the ‘Update My Information' link at the top of the page. For questions related to billing, please contact billing support.

If your institution or institutions have a press office, please notify them about your upcoming paper to help maximize its impact. If they’ll be preparing press materials, please inform our press team as soon as possible -- no later than 48 hours after receiving the formal acceptance. Your manuscript will remain under strict press embargo until 2 pm Eastern Time on the date of publication. For more information, please contact onepress@plos.org.

Kind regards,

Vijay Raghunathan

Academic Editor

PLOS One

Additional Editor Comments (optional):

Authors have revised the manuscript in a better way by considering the reviewer's comments . Also reviewer's have recommended acceptance. Hence manuscript is given accept decision.

Reviewers' comments:

Reviewer's Responses to Questions

**Comments to the Author**

1. If the authors have adequately addressed your comments raised in a previous round of review and you feel that this manuscript is now acceptable for publication, you may indicate that here to bypass the “Comments to the Author” section, enter your conflict of interest statement in the “Confidential to Editor” section, and submit your "Accept" recommendation.

Reviewer #1: All comments have been addressed

Reviewer #2: All comments have been addressed

**********

2. Is the manuscript technically sound, and do the data support the conclusions?

The manuscript must describe a technically sound piece of scientific research with data that supports the conclusions. Experiments must have been conducted rigorously, with appropriate controls, replication, and sample sizes. The conclusions must be drawn appropriately based on the data presented.

Reviewer #1: Yes

Reviewer #2: Yes

**********

3. Has the statistical analysis been performed appropriately and rigorously?

Reviewer #1: Yes

Reviewer #2: Yes

**********

4. Have the authors made all data underlying the findings in their manuscript fully available?

The PLOS Data policy requires authors to make all data underlying the findings described in their manuscript fully available without restriction, with rare exception (please refer to the Data Availability Statement in the manuscript PDF file). The data should be provided as part of the manuscript or its supporting information, or deposited to a public repository. For example, in addition to summary statistics, the data points behind means, medians and variance measures should be available. If there are restrictions on publicly sharing data—e.g. participant privacy or use of data from a third party—those must be specified.

Reviewer #1: Yes

Reviewer #2: Yes

**********

5. Is the manuscript presented in an intelligible fashion and written in standard English?

PLOS ONE does not copyedit accepted manuscripts, so the language in submitted articles must be clear, correct, and unambiguous. Any typographical or grammatical errors should be corrected at revision, so please note any specific errors here.

Reviewer #1: Yes

Reviewer #2: Yes

**********

6. Review Comments to the Author

Please use the space provided to explain your answers to the questions above. You may also include additional comments for the author, including concerns about dual publication, research ethics, or publication ethics. (Please upload your review as an attachment if it exceeds 20,000 characters)

Reviewer #1: The authors addressed all the comments very well and the manuscript can be accepted for publication.

Reviewer #2: all the best. The comments are addressed for satisfaction the present manuscript is Recommended for publication

**********

7. PLOS authors have the option to publish the peer review history of their article (what does this mean?). If published, this will include your full peer review and any attached files.

If you choose “no”, your identity will remain anonymous but your review may still be made public.

**Do you want your identity to be public for this peer review?**  For information about this choice, including consent withdrawal, please see our Privacy Policy.

Reviewer #1: No

Reviewer #2: No

**********

# Editor report: Acceptance letter

**DOI:** 10.1371/journal.pone.0352647.r004

**Authors:** Vijay Raghunathan

PONE-D-26-07993R1

PLOS One

Dear Dr. Son Minh,

I'm pleased to inform you that your manuscript has been deemed suitable for publication in PLOS One. Congratulations! Your manuscript is now being handed over to our production team.

At this stage, our production department will prepare your paper for publication. This includes ensuring the following:

* All references, tables, and figures are properly cited

* All relevant supporting information is included in the manuscript submission,

* There are no issues that prevent the paper from being properly typeset

You will receive further instructions from the production team, including instructions on how to review your proof when it is ready. Please keep in mind that we are working through a large volume of accepted articles, so please give us a few days to review your paper and let you know the next and final steps.

Lastly, if your institution or institutions have a press office, please let them know about your upcoming paper now to help maximize its impact. If they'll be preparing press materials, please inform our press team within the next 48 hours. Your manuscript will remain under strict press embargo until 2 pm Eastern Time on the date of publication. For more information, please contact onepress@plos.org.

You will receive an invoice from PLOS for your publication fee after your manuscript has reached the completed accept phase. If you receive an email requesting payment before acceptance or for any other service, this may be a phishing scheme. Learn how to identify phishing emails and protect your accounts at https://explore.plos.org/phishing.

If we can help with anything else, please email us at customercare@plos.org.

Thank you for submitting your work to PLOS ONE and supporting open access.

Kind regards,

PLOS ONE Editorial Office Staff

on behalf of

Dr. Vijay Raghunathan

Academic Editor

PLOS One
